# Seven-Membered Rings through Metal-Free Rearrangement Mediated by Hypervalent Iodine

**DOI:** 10.3390/molecules20011475

**Published:** 2015-01-15

**Authors:** Siguara Bastos Lemos Silva, Adriana Della Torre, João Ernesto de Carvalho, Ana Lúcia Tasca Gois Ruiz, Luiz F. Silva

**Affiliations:** 1 Departamento de Química Fundamental, Instituto de Química, Universidade de São Paulo, Av. Prof. Lineu Prestes, 748, CP 26077, São Paulo-SP CEP 05513-970, Brazil; E-Mail: siguara.silva@usp.br; 2 Division of Pharmacology and Toxicology, Multidisciplinary Center for Chemical, Biological and Agricultural, State University of Campinas, 6171, Campinas-SP CEP 13081-970, Brazil; E-Mails: adriana_biotec@yahoo.com.br (A.D.T.); carvalho_je@yahoo.com.br (J.E.C.); analucia@cpqba.unicamp.br (A.L.T.G.R.)

**Keywords:** hypervalent iodine, ring expansion, rearrangement, seven-membered ring, antiproliferative activity

## Abstract

A versatile and metal-free approach for the synthesis of carbocycles and of heterocycles bearing seven- and eight-membered rings is described. The strategy is based on ring expansion of 1-vinylcycloalkanols (or the corresponding silyl or methyl ether) mediated by the hypervalent iodine reagent HTIB (PhI(OH)OTs). Reaction conditions can be easily adjusted to give ring expansion products bearing different functional groups. A route to medium-ring lactones was also developed.

## 1. Introduction

**Figure 1:**
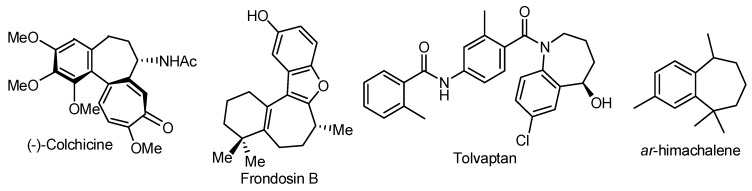
Compounds bearing seven-membered ring fused to aromatic ring.

The presence of seven-membered rings in compounds with remarkable biological activity continuously challenges organic chemists to develop efficient method for their preparation [1,2,3,4,5,6,7,8,9,10,11,12,13,14] (for examples of natural or designed compounds, see Figure 1). Construction of seven-membered rings is relatively more difficult than the corresponding process for five- and six-membered rings, mainly because cyclization reactions have the inherent drawback of entropic factors and transannular interactions [1,3,15]. Nevertheless, a variety of different methodologies were envisioned to circumvent these problems, such as palladium-catalyzed intramolecular reactions, and radical and electrophilic cyclizations [1,2,3,4,7,9,10,11,12]. Besides the palladium-catalyzed processes, other metal-mediated reactions were investigated and ring-closing metathesis and cycloadditions are probably the most used in the synthesis of seven- and eight-membered rings [5,6,7,8,9,10,12,16]. Another approach is a ring expansion reaction [12,17,18,19], which has the main advantage to avoid entropic factors and high-diluted conditions [1,15]. One possible strategy to promote a ring expansion is an oxidative rearrangement that can be performed with transition metals, such as palladium(II) [17], mercury(II) [20], and thallium(III) [21,22]. An alternative to prevent the use of these metals is a hypervalent iodine reagent that promotes several different reactions in an efficient manner, such as formation of C–C bonds, stereoselective oxidations, and many important functional group transformations, including asymmetric reactions [23,24,25,26,27,28,29,30]. Although oxidative rearrangements mediated by hypervalent iodine have been reported in many publications, systematic studies regarding ring expansion reactions are scarce [27]. The ring expansion of methylene derivatives mediated by PhI(OH)OTs (HTIB or Koser’s Reagent) has been investigated by Justik and Koser for the synthesis of six-, seven-, and eight-membered ring carbocyclic compounds [31,32]. This protocol was subsequently applied in the total synthesis of both isomers of *ar*-himachalene (Figure 1) [33]. This article presents a versatile and metal-free approach for the synthesis of molecules bearing a seven-membered ring, through a ring expansion reaction [34].

## 2. Results and Discussion

**Scheme 1:**
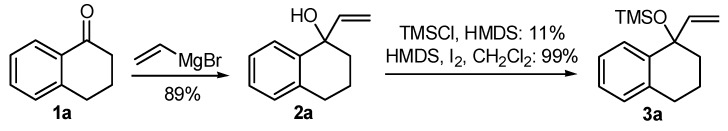
Preparation of the unsaturated TMS ether **3a**. HMDS: hexamethyldisilazane and TMSCl: trimethylsilylchloride.

The substrates required for the ring expansion reactions were prepared in an efficient manner. The reaction of 1-tetralone (**1a**) with CH_2_=CHMgBr gave the unsaturated 1-tetralol **2a**, in 89% yield [35]. Considering the possible instability of the tertiary benzylic and allylic alcohol **2a**, we decided to protect it as the trimethylsilyl (TMS) ether. The protocol using trimethylsilyl chloride/hexamethyldisilazane (TMSCl/HMDS) in reflux of hexane was applied to **2a**, giving the desired product **3a** in only 11% yield. However, using HMDS in the presence of a catalytic amount of I_2_, as reported by Karimi and Golshani [36], was possible to obtain cleanly **3a** in 99% yield (Scheme 1).

**Figure 2:**
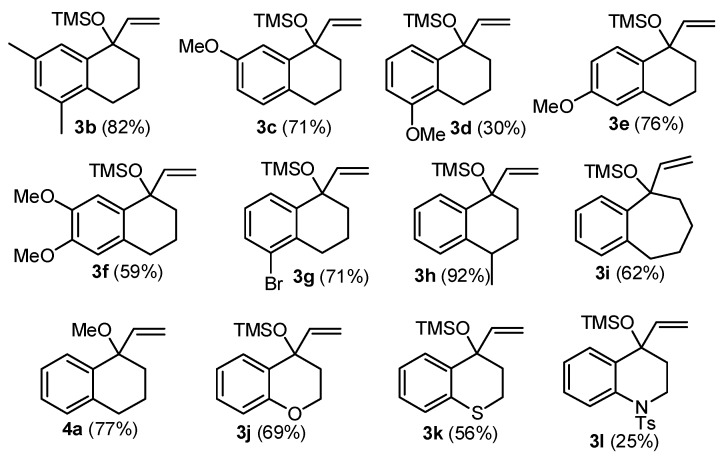
Structure of substrates **3b**–**l** and **4a**.

The above two-step sequence was applied to several ketones, leading to **3b-l**. We were also interested in the behavior of alkyl ethers. Thus, the methyl ether **4a** was prepared treating **2a** with KOH/MeI (Figure 2) [37].

**Scheme 2:**
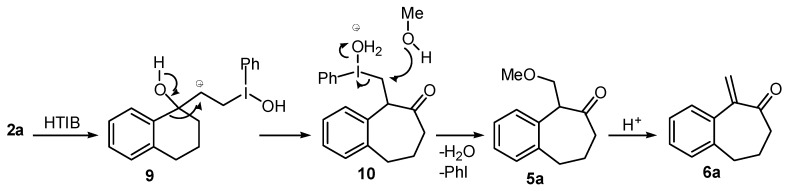
Mechanism of the Ring Expansion of **2a**. HTIB: [Hydroxy(tosyloxy)iodo]benzene.

We first performed a detailed investigation on the reactivity of the TMS-protected 1-vinylcycloalkanol **3a**. Thus, treatment of 3a with HTIB in CH_3_CN, in trimethylorthoformiate or without solvent [38] led to a complex mixture of compounds. Fortunately, when the unsaturated TMS-ether **3a** was treated with HTIB in MeOH [31] in the presence of *p*-TsOH, thin layer chromatography (TLC) analysis indicated the cleavage of the labile TMS-group. Then, the alcohol **2a** formed in the medium reacted with iodine(III), giving the ring expansion product 5a, in 60% yield (Table 1, Entry 1). The methoxy-ketone **5a** would be originated from **3a** in four steps. The first would be the acid-catalyzed deprotection of the TMS group, giving **2a**, on which the electrophilic addition of iodine(III) to the double bond would give the cation **9**. Migration of the aryl carbon would lead to **10**. A reductive solvolysis on **10** would produce the methoxylated ketone **5a** (Scheme 2). On this step occurs the highly favorable transformation of the hypervalent iodine into the normal valency compound PhI. Higher temperatures and longer reaction times promote an acid-catalyzed elimination of MeOH from **5a**, furnishing the enone **6a**, together with the dimer **7a** (entry 2). TLC analysis showed that **7a** is formed after the work-up. This result is slightly different from that using Tl(III), which gives only the enone **6a** from **3a** [22].

**Table 1:** HTIB-Mediated Ring Expansion of **3a**. HTIB: [Hydroxy(tosyloxy)iodo]benzene; *p*-TsOH: *p*-Toluenesulfonic acid.

Entry	Conditions	Product (Yield)
1	1.0 equiv HTIB, 20 mol% *p*-TsOH, MeOH, −72 °C to rt, 2 h	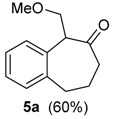
2	1.0 equiv HTIB, MeOH, −72 to 30 °C, 2.5 h	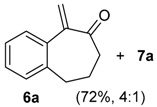
3	(1) 1.0 equiv HTIB, MeOH, −72 to 30 °C, 2.5 h; (2) 2 weeks	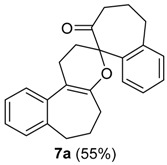
4	2.5 equiv HTIB, MeOH, rt, 2 h	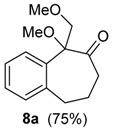

**Scheme 3:**
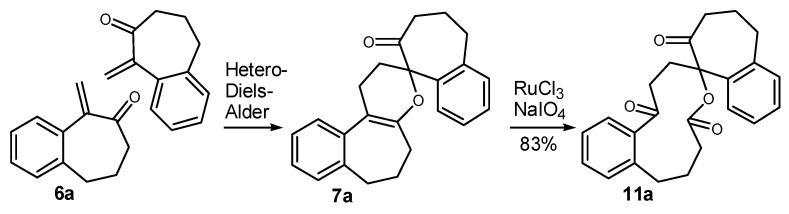
Preparation of the Medium-Ring Keto-Lactone **11a**.

On standing, the mixture **6a/7a** gave pure crystals of **7a**, in 55% yield from **3a** (Table 1, entry 3), whose structure was assigned by X-ray analysis [34]. The pentacyclic compound **7a** is formed from the 1-vinylcycloalkanol derivative **3a** in a single operation through a tandem ring-expansion/hetero-Diels-Alder reaction [39,40]. We envisioned that **7a** could be used to obtain a medium ring lactone [41,42]. Indeed, the oxidative cleavage of the double bond of **7a** could be performed with RuCl_3_/NaIO_4_, giving the eleven-membered ring keto-lactone **11a** (Scheme 3). In summary, the commercially available 1-tetralone (**1a**) was transformed in only four steps into **11a**, which bears a spiro seven-membered ring and a medium-ring lactone. Thus, in this short sequence of steps, the molecular complexity is greatly increased, because several reactions took place in a few operations.

Since the double bond of enone **6a** is prone to further oxidation, we decided to investigate the reaction of **3a** with excess of oxidant. When **3a** was treated with 2.5 equiv of HTIB, a tandem ring expansion/addition of MeOH gave the dimethoxy-ketone **8a** (Table 1, entry 4). An iodine(III)-mediated electrophilic addition of MeOH to the enone **6a** would give **8a**. In summary, different ring expansion products **5a**–**8a** can be obtained from the same substrate (**3a**) by modification of the reaction conditions.

**Scheme 4:**
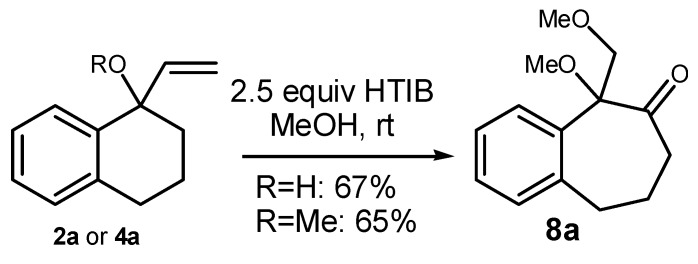
Tandem Expansion/Addition of **2a** and **4a** by HTIB. HTIB: [Hydroxy(tosyloxy)iodo]benzene.

After exploring the oxidation of **3a** with iodine(III) under several conditions, we checked if the protection as a silyl ether was really required. The desired dimethoxy-ketone **8a** was also obtained when either **2a** or **4a** were treated with HTIB (Scheme 4). In conclusion, the presence of the TMS group is not essential for the ring expansion, although higher yields of the desired product were observed from **3a** (75%) than from **2a** or from **4a** (65%–67%). However, the protection of the tertiary benzylic and allylic alcohol **3a **as a TMS ether greatly facilitate the storage of the substrate and we decide to do this for all substrates.

A substituent in the aromatic ring can alter the migratory aptitude of the migrating carbon, which may influence the yield of the rearrangement product. For example, a correlation between yield of the product and migratory aptitude was noted by us in Tl(III)-mediated ring contraction of 1,2-di-hydronaphthalenes [43]. Thus, we investigated the ring expansion of **3** with different groups in the aromatic ring. Alkyl groups in the aromatic ring can be problematic in reactions with hypervalent iodine [44,45]. Fortunately, the TMS-protected alcohol **3b**, which bears methyl groups, gave the dimethoxy ketone **8b** (Table 2, Entry 1) in a similar yield to the non-substituted substrate **3a**. A methoxy group at the *meta* position could decrease the migratory aptitude of the migrating carbon. The value of the Hammett constant ρ_m_ for OMe is 0.11. Hence, a lower yield of the ring expansion product could be expected. However, the reaction of **3c**–**d** with HTIB led to the corresponding ring expansion products **8c**–**d**, respectively, in comparable yield (Entries 2 and 3). A methoxy group in the *para* position of the migrating carbon increases the migratory aptitude, which could accelerate the rearrangement. In our experience, this is usually a beneficial effect [43,46]. However, the reaction with **3e** gave the ring expansion product **8e**, in only 10% yield (entry 4). After some experimentation, we found that treating **3e** with HTIB in a mixture of AcOEt/MeOH gave **8e**, in 67% yield (Entry 5).

**Table 2:** HTIB-Promoted Tandem Ring Expansion/Addition in MeOH. HTIB: [Hydroxy(tosyloxy)iodo]benzene; TMS: trimethylsilyl.

Entry	Substrate	Products (Isolated Yield)
1	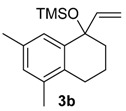	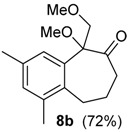
2	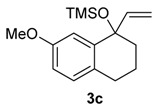	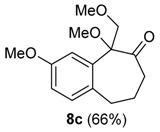
3	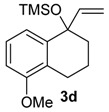	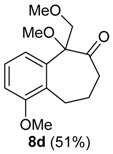
4	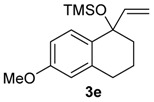	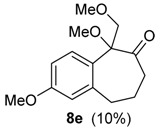
5 ^a^	**3e**	**8e** (67%)
6 ^a^	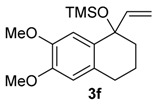	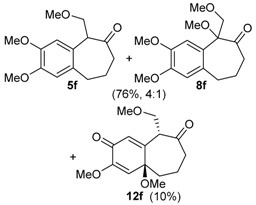
7 ^b^	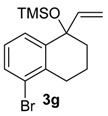	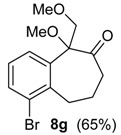
8	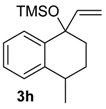	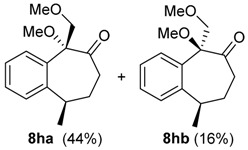
9	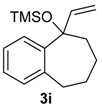	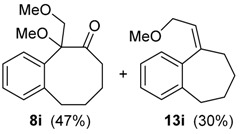
10	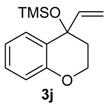	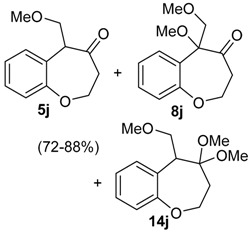
11	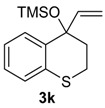	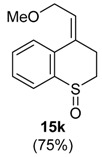
12 ^b^	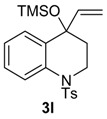	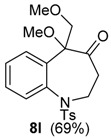

^a^ AcOEt/MeOH (2:1), −72 °C–rt; ^b^ MeOH, 0–50 °C.

**Scheme 5:**
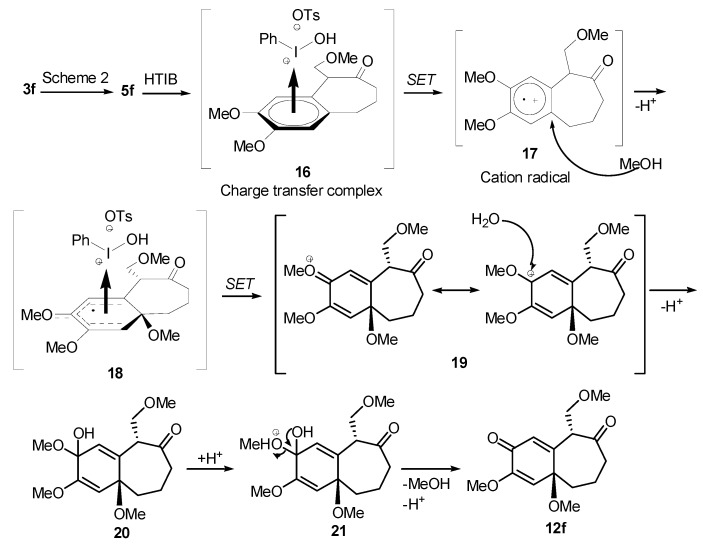
Mechanism for the Formation of **12f**.

**Figure 3:**
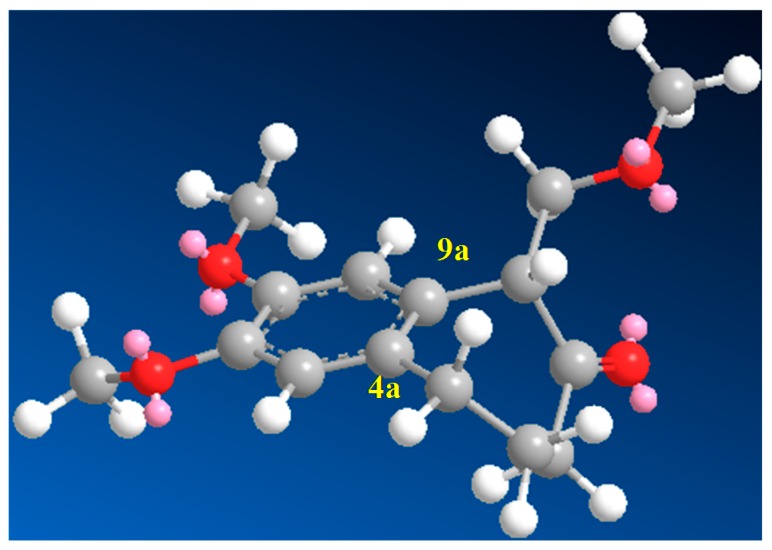
Structure of **17**.

The same solvent mixture (AcOEt/MeOH) was also used in the reaction of **3f**. In this case, a mixture of the seven-membered ring compounds **5f**, **8f** and **12f** were isolated in very good overall yield (Entry 6). Compounds **5f** and **8f** could not be separated from each other by chromatography column or HPLC. The proposed mechanism for the formation of **12f** was based on desaromatization reactions previously described in literature [47,48] (Scheme 5). The first step is the transformation of **3f** into the seven-membered ring compound **5f**, as shown in (Scheme 2), followed by the formation of the charge transfer complex **16** from **5f** and HTIB. A single-electron-transfer (SET) oxidation of **16** yields the cation radical **17**. Species **17** suffers a MeOH attack from the less hindered convex face and at less hindered carbon 4a (Figure 3), giving the radical **18**. A second SET leads to carbocation **19**, which reacts with the solvent yielding **20**. The enone **12f **is formed after an acid hydrolysis of **20** catalyzed by acid. The relative configuration of **12f** was assigned by NMR analysis, including NOESY, HMBC and HSQC (see Supplementary Informationfor details). 

**Scheme 6:**
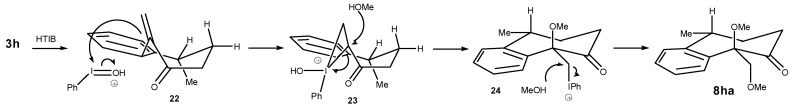
Mechanism for the Formation of **8ha**.

The reaction of the bromo-substituted substrate 3 g with HTIB needed heating until 50 °C to furnish the ring expanded product in good yield (Table 2, Entry 7). As expected, a withdrawing group as bromide in *meta* position to migrating carbon decreases its aptitude to migration and, thus, more energetic conditions were necessary. Substrate 3h was exposure to HTIB giving 8 ha/b in 44 and 16%, respectively (Entry 8). The stereoselectivity is determined in the electrophilic addition of iodine(III) to the enone 22. This step occurs preferentially through the less hindered face (Scheme 6).

**Scheme 7:**
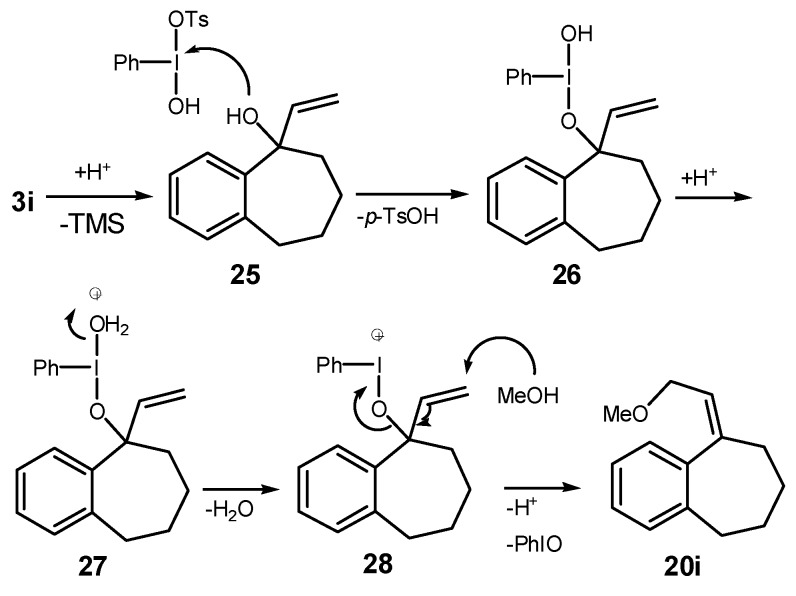
Mechanism for the Formation of **20i**.

The possibility of using a ring expansion reaction to prepare eight-membered rings was also investigated. Substrate **3i** was treated with HTIB, giving the desired eight-membered ring compound **8i** in 47% yield, together with the unsaturated ether **13i** (entry 9). The relative configuration of **13i** was assigned based on NMR data of related compounds [49]. This route can be useful to obtain eight-membered ring derivatives, because only three steps are necessary to obtain **8i** from the commercially available benzosuberone. The first step in the formation of **20i** (Scheme 7) is a ligand exchange from HTIB with **25**, giving **26**. A sequence of protonation of **26** and dehydration of **27** lead to **28**, that participates in a S_N_2’ reaction with the solvent, yielding **20i**.

The reactivity of heterocyclic substrates was also examined. When compound **3j** was treated with HTIB, the ring expansion reaction also took place. However, an inseparable mixture of seven-membered ring *O*-heterocycles**5j**, **8j**, and **14j** was isolated (Table 2, Entry 10). The oxygen at the *ortho* position of the migrating carbon **8j** change the reactivity, as observed in other oxidative rearrangements promoted by iodine(III) [50]. Treatment of the sulfur derivative **3k** with HTIB gave exclusively the sulfoxide, in 75% yield (Table 2, entry 11). The first reaction is the oxidation of the sulfide moiety to the corresponding sulfoxide [51]. This electron-withdrawing group would decrease the migratory aptitude of the migrating carbon and the S_N_2’ reaction became the favorable pathway. The reaction of substrate **3l** with HTIB furnished the benzazepine **8l** in good yield (Table 2, Entry 12). Structures like **8l** are present in many natural products [52] and have different biological activities [53,54,55,56], being important building blocks for drugs. Among the methodologies for the preparation of benzazepines [57,58,59,60,61,62], metals are involved in most of them and a metal free approach could be a useful alternative, specially for pharmaceuticals applications.

The antiproliferative activity of seven-membered rings products (**5f** + **8f**, **8d**, **8g**, **12f** and **8l**) was evaluated against a panel of nine human tumor cell lines and one immortalized human cell line using a protocol described in the literature [63,64]. This methodology aims to evaluate a group of samples in many different tumor cell lines to find evidence of their antiproliferative profile. In order to prioritize further evaluations, a threshold for mean logTGI (Total Growth Inhibition) values (mean log TGI ≤ 1.50) was assumed [65]. 

Compounds **5f** + **8f**, **8g** and **8l** can be classified as inactive considering the average antiproliferative effect (mean logTGI > 1.50) (Table 3). The mixture **5f** + **8f** (1:1) showed a selective growth inhibitory effect against glioma (U251, TGI = 4.8 µg·mL^−1^) and prostate (PC-3, TGI = 3.6 µg·mL^−1^) cell lines. Moreover, compounds **12f** and **8d** showed, respectively, a moderate (mean logTGI = 1.03) and a weak (mean logTGI = 1.35) citostatic effects. This suggests that the presence of methoxy groups in the ring fused to the seven-membered system can contribute to the antiproliferative activity and the inclusion of a methoxy group on the carbon of the ring fusion can increase this effect.

**Table 3:** Antiproliferative activities (TGI, µg·mL^−1^) of ring expansion products ^a^.

	Doxorubicin	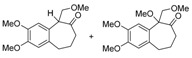	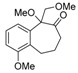	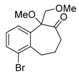	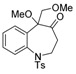	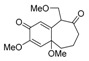
		5f and 8f (1:1)	8d	8g	8l	12f
U251	0.20	4.8	8.2	43.2	50.7	5.2
UACC-62	0.86	18.1	34.4	59.3	91.3	8.4
MCF-7	1.2	14.0	25.9	52.8	70.1	6.0
NCI-ADR/RES	3.5	19.8	42.0	57.9	79.9	6.5
786-0	0.27	15.9	27.6	72.8	102.4	6.7
NCI-H460	0.61	37.2	60.3	64.6	157.7	22.9
PC-3	0.74	3.6	8.5	44.8	88.5	10.6
HT29	11.4	17.5	24.4	55.7	124.6	34.6
K562	0.96	>250	25.6	>250	167.6	46.3
HaCat	0.16	7.5	11.8	48.1	206.0	4.1
Mean LogTGI	−0.081	>1.2	1.35	>1.8	2.02	1.03

^a^ Tumor human cell lines: U251 (glioma); UACC-62 (melanoma); MCF-7 (breast); NCI-ADR/RES (ovarian resistant to multiple drugs); 786-0 (kidney); NCI-H460 (lung, non small cells); PC-3 (prostate); HT29 (colon); K562 (leukemia). Immortalized non-tumoral cell line: HaCat (human keratinocyte).

## 3. Experimental Section

### General Information 

HTIB, HMDS and MeOH were used as received. THF (tetrahydrofuran) was freshly distilled from sodium/benzophenone, CH_2_Cl_2_ was distilled from CaH_2_ and stored with molecular sieves 3 Å. Vinyl magnesium bromide was purchased from Aldrich or prepared from vinyl bromide and magnesium turnings [66]. 1-Tetralone was distilled before used (bp: ~155 °C, 32 mmHg). Column chromatography was performed using silica gel 200–400 Mesh. TLC analyses were performed in silica gel 60 F_254_ plates, using UV, I_2_, *p*-anisaldehyde, or phosphomolybdic acid solution for visualization. ^1^H- and ^13^C-NMR spectra were recorded on Bruker (Billerica, MA, USA) or Varian spectrometers (Palo Alto, CA, USA). IR spectra were measured on a Perkin-Elmer 1750-FT (Waltham, MA, USA). Gas chromatography analyses were performed in a HP-6890 series II (Agilent, Santa Clara, CA, USA) and/or Shimadzu-2010 (Kyoto, Japan). Melting points were done in Büchi Melting Point B-545 (Flawil, Switzerland) and are uncorrected. HRMS analyses were performed on a Bruker Daltonics Microtof Eletrospray (Billerica, MA, USA). CHN analyses were performed with Perkin-Elmer CHN 2400 equipment (Waltham, MA, USA). The percentage of bromine in the organic compounds was determined by volumetric titration using a solution of Hg(NO_3_)_2_ and diphenylcarbazone as indicator. The tests with KI paper were performed applying a drop of the reaction mixture in a filter paper previously impregnated with a solution of KI (10%), which was dried at 100 °C. The preparation of compounds **3b**–**c** and **3h**–**k** was reported in the previous communication [34].

*1-Tosyl-2,3-dihydroquinolin-4(1H)-one* (**1l**). To a mixture of 1,2,3,4-tetrahydroquinoline (3.7 mL, 4.0 g, 30 mmol) in anhydrous pyridine (15 mL), was added TsCl (7.63 g, 40.0 mmol, 1.3 equiv) at rt. The mixture was stirred at 60 °C for 15.5 h. The temperature was increased to 110 °C and the mixture was stirred for 5.5 h. The reaction mixture was cooled to −5 °C and hot H_2_O (25 mL) was added, precipitating the crude product, which was filter under reduced pressure. The solid was washed with HCl (0.01 mol·L^−^^1^) and H_2_O, and dried in the air. The small and brownish crystals (9.808 g) were recrystallized with MeOH (200 mL), giving colorless crystals of 1-tosyl-1,2,3,4-tetrahydroquinoline [67] (7.15 g, 24.9 mmol, 83%). mp: 94.6–95.2 °C (lit.[67]: 91–92 °C).To a solution of 1-tosyl-1,2,3,4-tetrahydroquinoline (1.56 g, 5.00 mmol) in acetone (22.5 mL) at 0 °C was added anhydrous MgSO_4_ (1.51 g, 12.5 mmol, 2.5 equiv) and H_2_O (9.0 mL). Subsequently, KMnO_4_ (4.35 g, 27.5 mmol, 5.5 equiv) was added dropwise for 30 min. The mixture was stirred for 27 h at rt. The solid was filtered under reduced pressure, washed with CH_2_Cl_2_ and H_2_O. Saturated solution of K_2_S_2_O_5_ (50 mL) was added to the resulting solution. The solid was filtered under reduced pressure. The solution was extracted with CH_2_Cl_2_, washed with brine, and dried with anhydrous MgSO_4_. The solvent was removed under reduced pressure, giving **1l** [68], as white crystals (1.18 g, 3.93 mmol, 79%). mp 141.1–141.9 °C (lit. [69] 141–142 °C).

*5-Methoxy-1-vinyl-1,2,3,4-tetrahydronaphthalen-1-ol* (**2d**). General Procedure for the Preparation of Allylic Alcohols. To a solution of 5-methoxy-1-tetralone (3.52 g, 20.0 mmol) in anhydrous THF (20 mL) in a Schlenk flask, was added CH_2_=CHMgBr in THF (1 mol·L^−1^, 50.0 mL, 50.0 mmol) at 0 °C. The mixture was stirred for 3–4 h at rt. Saturated solution of NH_4_Cl (32 mL) was added dropwise at 0 °C. The aqueous phase extracted with AcOEt, dried under anhydrous MgSO_4_, filtered, and the solvent was removed under reduced pressure. The crude product was purified by flash column chromatography (hexanes/AcOEt, 4:1), giving **2d** (2.97 g, 14.6 mmol, 73%), as colorless oil. IR (film) ν/cm^−1^ 1257, 1467, 1583, 2938, 3430; ^1^H-NMR (300 MHz, CDCl_3_) δ 1.79–1.98 (m, 5H), 2.53–2.83 (m, 2H), 3.82 (s, 3H), 5.19 (dd, 1H, *J =* 1.7, 10.8), 5.29 (dd, 1H, *J* = 1.7, 17.1), 6.04 (dd, 1H, *J =* 10.8, 17.1), 6.75 (dd, 1H, *J =* 0.9, 8.1), 7.00–7.03 (m, 1H), 7.17 (t, 1H, *J =* 8.1); ^13^C-NMR (75 MHz, CDCl_3_) δ 18.5, 23.2, 37.3, 55.4, 73.3, 108.5, 113.1, 119.7, 126.2, 126.4, 141.0, 144.8, 156.9; LRMS *m/z* (%) 63 (18), 115 (98), 128 (54), 141 (58), 155 (65), 171 (55), 186 (100); HRMS (ESI) *m/z*, calcd for [C_13_H_16_O_2_+Na]^+^ 227.1048, found: 227.1039.

*((5-Methoxy-1-vinyl-1,2,3,4-tetrahydronaphthalen-1-yl)oxy)trimethylsilane* (**3d**). General Procedure for the Protection with a TMS group. A solution of **2d** (2.86 g, 14.0 mmol), I_2_ (a crystal) in anhydrous CH_2_Cl_2_ (56 mL) was added dropwise for 5 min to a solution of HMDS (2.4 mL, 11 mmol) in anhydrous CH_2_Cl_2_ (14 mL). This mixture was stirred for 30 min at rt and Na_2_S_2_O_3_ (4.2 g) was added. The mixture became clear and was stirred for 30 min. The mixture was filtered through a silica pad (5 × 2 cm) using CH_2_Cl_2_ as eluent. The solvent was removed under reduced pressure and the product was purified by flash column chromatography (hexanes/AcOEt, 17:3), giving **3d** (1.60 g, 5.79 mmol, 41%) as a slightly yellow oil. IR (film) ν/cm^−1^ 837, 1046, 1257, 1457, 1584, 2941; ^1^H-NMR (300 MHz, CDCl_3_) δ −0.04 (s, 9H), 1.70–1.85 (m, 1H), 1.87–1.99 (m, 3H), 2.56–2.78 (m, 2H), 3.81 (s, 3H), 5.05 (dd, 1H, *J =* 1.8, 16.8), 5.09 (dd, 1H, *J =* 1.8, 10.5), 6.04 (dd, 1H, *J =* 10.5, 16.8), 6.71 (dd, 1H, *J =* 1.2, 7.8), 7.04 (dd, 1H, *J =* 1.2, 7.8), 7.13 (t, 1H, *J =* 7.8); ^13^C-NMR (75 MHz, CDCl_3_) δ 2.2, 18.9, 23.0, 37.4, 55.3, 76.4, 108.1, 113.0, 120.9, 125.7, 126.1, 141.4, 145.7, 156.6; LRMS *m/z* (%) 73 (100), 115 (23), 128 (17), 158 (34), 171 (17), 186 (54), 276 (M^+•^, 11), 248 (40); HRMS (ESI) *m/z*, calcd for [C_16_H_24_O_2_Si+Na]^+^: 299.1443, found: 299.1442.

*((6,7-Dimethoxy-1-vinyl-1,2,3,4-tetrahydronaphthalen-1-yl)oxy)trimethylsilane* (**3f**). The general procedure was followed using 6,7-dimethoxy-1-tetralone (2.12 g, 10.0 mmol), THF (17 mL), and CH_2_=CHMgBr (1 M in THF, 27.2 mL, 27.2 mmol). A solution of the crude product (2.69 g) in anhydrous CH_2_Cl_2_ (10 mL) was added dropwise for 5 min to a solution of HMDS (1.7 mL, 8.4 mmol) and of I_2_ (a crystal) in anhydrous CH_2_Cl_2_ (40 mL). This mixture was stirred for 30 min at rt and Na_2_S_2_O_3_ (3.11 g) was added. The mixture became clear and was stirred for 30 min. The mixture was filtered through a silica pad (5 × 2 cm) using CH_2_Cl_2_ as eluent. The solvent was removed under reduced pressure and the product was purified by flash column chromatography (hexanes/AcOEt, 3:2), giving **3f**, as a white solid (1.81 g, 5.91 mmol, 59%). mp 48.5–49.7 °C; IR (film) ν/cm^−1^ 840, 910, 928, 1032, 1049, 1116, 1137, 1216, 1261, 1462, 1516, 2952, 3001; ^1^H-NMR (300 MHz, CDCl_3_) δ −0.03 (s, 9H), 1.71–1.84 (m, 1H), 1.87–1.98 (m, 3H), 2.62–2.79 (m, 2H), 3.83 (s, 3H), 3.85 (s, 3H), 5.06 (dd, 1H, *J =* 1.8, 16.8), 5.09 (dd, 1H, *J =* 1.8, 10.5), 6.02 (dd, 1H, *J =* 10.5, 16.8), 6.52 (s, 1H), 6.89 (s, 1H); ^13^C NMR (75 MHz, CDCl_3_) δ 2.2, 19.8, 29.1, 38.0, 55.7 (2C), 76.4, 110.7, 111.5, 112.9, 129.4, 132.0, 145.8, 146.8, 148.1; anal. calcd for C_17_H_26_O_3_Si: C, 66.62; H, 8.55, found: C, 67.03; H, 8.69 (% H); LRMS *m/z* (%) 45 (41), 73 (100), 115 (21), 128 (16), 188 (16), 216 (17), 179 (39), 306 (M^+•^, 6); HRMS (ESI) *m/z*, calcd for [C_17_H_26_O_3_Si+Na]^+^: 329.1549, found: 329.1552.

*((5-Bromo-1-vinyl-1,2,3,4-tetrahydronaphthalen-1-yl)oxy)trimethylsilane* (**3g**). The general procedure was followed using 5-bromo-1-tetralone (0.788 g, 3.50 mmol), THF (25 mL), and CH_2_=CHMgBr (1 M in THF, 8.8 mL, 8.8 mmol, 2.5 equiv). The crude product was protected with a TMS group following the general procedure, but using HMDS (0.7 mL, 3.2 mmol) in CH_2_Cl_2_ (4 mL), and a solution of the crude alcohol (1.01 g) and I_2_ (a crystal) in CH_2_Cl_2_ (16 mL). The mixture was filtered through a silica pad (5 × 2 cm) using hexanes/Et_2_O (97:3) as eluent, giving **3g** (0.783 g, 2.50 mmol, 71%), as slightly yellow oil. IR (film) ν/cm^−1^ 756, 840, 900, 915, 1048, 1251, 2948; ^1^H-NMR (300 MHz, CDCl_3_) δ −0.02 (s, 9H), 1.77–1.86 (m, 1H), 1.89–2.01 (m, 3H), 2.67–2.82 (m, 2H), 4.99 (dd, 1H, *J =* 1.5, 17.1), 5.12 (dd, 1H, *J =* 1.5, 10.5), 6.02 (dd, 1H, *J =* 10.5, 17.1), 7.03 (t, 1H, *J =* 7.8), 7.40 (dd, 1H, *J =* 1.2, 7.8), 7.44 (dd, 1H, *J =* 1.2, 7.8); ^13^C-NMR (75 MHz, CDCl_3_) δ 2.2, 19.2, 30.3, 37.1, 77.2, 113.9, 124,9, 126.7, 128.1, 131.3, 136.4, 143.0, 145.3; anal. calcd for C_15_H_21_BrOSi: C, 55.38; H, 6.51; Br, 24.56, found: C, 55.61; H, 6.55; Br, 24.67; LRMS *m/z* (%) 45 (33), 73 (100), 115 (21), 128 (21), 155 (34), 296/298 (26), 326/324 (M^+•^, 6). 

*1-Tosyl-4-((trimethylsilyl)oxy)-4-vinyl-1,2,3,4-tetrahydroquinoline* (**3l**). The general procedure was followed using **1l** (2.41 g, 8.00 mmol), THF (25 mL), and CH_2_=CHMgBr (1 M in THF, 20.0 mL, 20.0 mmol, 2.5 equiv). The crude alcohol was purified by flash column chromatography (CH_2_Cl_2_ as eluent), giving the alcohol (0.631 g, 2.00 mmol, 25%), as yellow oil. The alcohol was protected following the general procedure, but using HMDS (0.34 mL, 1.6 mmol, 0.8 equiv) in CH_2_Cl_2_ (2 mL) and a solution of the alcohol (0.631 g, 2.00 mmol) and I_2_ (a crystal) in CH_2_Cl_2_ (8 mL). Compound **3l** (0.800 g, 1.99 mmol, 100%) was obtained as brown oil. IR (film) ν/cm^−1^ 840, 1166, 1357, 2957; ^1^H-NMR (300 MHz, CDCl_3_) δ −0.13 (s, 9H), 1.64–1.82 (m, 2H), 2.37 (s, 3H), 3.86–4.01 (m, 2H), 4.89 (dd, 1H, *J =* 1.5, 16.8), 5.00 (dd, 1H, *J =* 1.5, 10.5), 5.66 (dd, 1H, *J =* 10.5, 16.8), 7.08 (td, 1H, *J =* 1.2, 7.5, 11.7), 7.19–7.22 (m, 2H), 7.23–7.27 (m, 1H), 7.32 (dd, 1H, *J =* 1.8, 11.7), 7.55 (dt, 2H, *J =* 1.8, 8.4), 7.87 (dd, 1H, *J =* 0.9, 8.4); ^13^C-NMR (75 MHz, CDCl_3_) δ 2.2, 21.5, 35.2, 43.4, 73.8, 114.4, 122.6, 124.1, 127.2 (2C), 128.3, 129.4, 129.6 (2C), 131.8, 136.1, 136.7, 143.7, 143.8; LRMS *m/z* (%) 45 (10), 73 (54), 91 (27), 130 (20), 156 (100), 218 (9), 246 (18), 401 (M^+•^, 3); HRMS (ESI) *m/z*, calcd for [C_21_H_27_NO_3_SSi+Na]^+^: 424.1379, found: 424.1376.

*1,5-Dimethoxy-5-(methoxymethyl)-5,7,8,9-tetrahydro-6H-benzo*[7]*annulen-6-one* (**8d**). General Procedure for the Ring Expansion of TMS-protected Allylic Alcohols. To a solution of **3d** (0.138 g, 0.500 mmol) in MeOH (2 mL) was added HTIB (0.490 g, 1.25 mmol) at 0 °C. The progress of the reaction was monitored by filter paper impregnated with a solution of KI (10%). The reaction was stirred for 1 h at this temperature and 1 h at rt. The reaction was quenched with saturated solution of NaHCO_3_ (3 mL). The aqueous phase was extracted with CH_2_Cl_2_ (3 × 5 mL). The organic phase was washed with H_2_O and with brine. The organic phase was dried with anhydrous MgSO_4_, filtered and the solvent was removed under reduced pressure. The residue was purified by flash column chromatography (hexanes/Et_2_O, 2:3), giving 8d (0.0676 g, 0.256 mmol, 51%), as yellow oil. IR (film) ν/cm^−1^ 1079, 1098, 1469, 1581, 1718, 2834, 2935; ^1^H-NMR (300 MHz, CDCl_3_) δ 1.67–1.82 (m, 1H), 2.06–2.18 (m, 1H), 2.40–2.47 (m, 1H), 2.96 (dtd, 1H, *J =* 3.3, 13.2), 3.19 (s, 3H), 3.34–3.20 (m, 2H), 3.39 (s, 3H), 3.81 (s, 3H), 3.98 (d, 1H, *J =* 9.6), 4.21 (d, 1H, *J =* 9.6), 6.83–6.89 (m, 2H), 7.19 (t, 1H, *J =* 8.1); ^13^C-NMR (75 MHz, CDCl_3_) δ 21.6, 27.5, 39.5, 51.2, 55.9, 59.7, 72.4, 87.6, 111.3, 119.3, 127.0, 129.9, 136.2, 157.0, 210.1; LRMS *m/z* (%) 45 (56), 77 (22), 91 (29), 115 (25), 144 (41), 159 (61), 172 (37), 191 (100), 219 (36), 264 (M^+•^, 5); HRMS (ESI) *m/z*, calcd for [C_15_H_20_O_4_+Na]^+^: 287.1259, found: 287.1258.

*Oxidation of* **3f** *with HTIB*. The general procedure for ring expansion was followed, using HTIB (0.490 g, 1.25 mmol), solution of **3f** (0.153 g, 0.500 mmol) and of PTSA (0.020 g, 0.12 mmol, 20 mol %) in AcOEt/MeOH (2:1, 3 mL) at −72 °C. The residue was purified by flash column chromatography (hexanes/AcOEt, 3:7), giving a mixture of **5f** and **8f** (0.100 g, 0.424 mmol, 76%), as yellow oil and **12f** (0.015 g, 0.054 mmol, 10%), as white solid. 2,3-Dimethoxy-5-(methoxymethyl)-5,7,8,9-tetrahydro-6*H*-benzo[7]annulen-6-one (**5f**): ^1^H-NMR (500 MHz, CDCl_3_) δ 1.88–1.95 (m, 1H), 2.05–2.11 (m, 1H), 2.54–2.58 (m, 1H), 2.67–2.72 (m, 1H), 2.84–2.94 (m, 2H), 3.38 (s, 3H), 3.83 (dd, 1H, *J =* 6.0, 9.0), 3.87 (d, 6H, *J =* 2.0), 4.00–4.02 (m, 1H), 4.12 (dd, 1H, *J =* 7.5, 9.0), 6.64 (s, 1H), 6.71 (s, 1H); ^13^C-NMR (125 MHz, CDCl_3_) δ 28.0, 32.6, 43.5, 55.9, 56.1, 56.6, 59.2, 71.2, 111.5, 113.1, 126.6, 133.0, 147.6, 147.8, 209.3; HRMS (ESI) *m/z*, calcd for [C_15_H_20_O_4_+Na]^+^: 287.1259, found: 287.1259. 2,3,5-Trimethoxy-5-(methoxymethyl)-5,7,8,9-tetrahydro-6*H*-benzo[7]annulen-6-one (**8f**): ^1^H-NMR (500 Hz, CDCl_3_) δ 1.80–1.87 (m, 1H), 2.12–2.19 (m, 1H), 2.44 (quin, 1H, *J =* 5.5), 2.63 (ddd, 1H, *J =* 3.5, 6.5, 14.0), 3.19 (s, 3H), 3.24–3.32 (m, 2H), 3.42 (s, 3H), 3.88 (d, 6H*, J =* 2.0), 3.98 (d, 1H, *J =* 10.0), 4.20 (d, 1H, *J =* 9.5), 6.62 (s, 1H), 6.67 (s, 1H); ^13^C-NMR (125 MHz, CDCl_3_) δ 28.9, 33.2, 39.4, 51.0, 55.8, 56.1, 59.7, 72.1, 87.5, 110.8, 114.2, 126.3, 134.5, 147.3, 148.6, 209.9; HRMS (ESI) *m/z*, calcd for [C_16_H_22_O_5_+Na]^+^: 317.1365, found: 317.1364. *trans-*3,4a-Dimethoxy-9-(methoxymethyl)-4a,5,6,7-tetrahydro-2*H*-benzo[7]annulene-2,8(9*H*)-dione (**12f**): mp 160.4–161.0 °C; IR (film) ν/cm^−1^ 1091, 1170, 1227, 1392, 1451, 1669, 1700, 2937; ^1^H-NMR (300 MHz, CDCl_3_) δ 1.46–1.55 (m, 1H), 1.67–1.76 (m, 1H), 2.30–2.60 (m, 4H), 3.62 (dd, 1H, *J =* 7.2, 9.6), 3.71 (s, 3H), 3.98 (dd, 1H, *J =* 6.6, 9.6), 4.34 (t, 1H, *J =* 6.9), 5.64 (s, 1H), 6.23 (d, 1H, *J =* 0.9); ^13^C-NMR (75 MHz, CDCl_3_) δ 17.8, 42.6, 42.9, 50.9, 52.6, 55.1, 59.2, 69.9, 77.4, 118.7, 128.6, 151.7, 158.0, 180.1, 205.1; LRMS *m/z* (%) 39 (49), 51 (76), 65 (44), 77 (84), 91 (100), 103 (42), 115 (57), 131 (42), 149 (51), 161 (54), 177 (65), 192 (40), 205 (60), 220 (96), 233 (26), 248 (43); HRMS (ESI) *m/z*, calcd for [C_15_H_20_O_5_+Na]^+^: 303.1208, found: 303.1207.

*1-Bromo-5-methoxy-5-(methoxymethyl)-5,7,8,9-tetrahydro-6H-benzo*[7]*annulen-6-one* (**8g**). To a solution of **3g** (0.193 g, 0.481 mmol) and of PTSA (0.020 g, 0.12 mmol, 24 mol %) in MeOH (3 mL) was added HTIB (0.471 g, 1.20 mmol) at 0 °C. The mixture was stirred for 1 h. The temperature was increased to rt and the mixture was stirred for 2 h. The temperature was increased to 50 °C and the mixture was stirred for 3 h. The reaction was quenched with saturated solution of NaHCO_3_ (5 mL). The aqueous phase was extracted with CH_2_Cl_2_ (3 × 5 mL). The organic phase was washed with H_2_O and with brine. The organic phase was dried with anhydrous MgSO_4_, filtered and the solvent was removed under reduced pressure. The residue was purified by flash column chromatography (hexanes/AcOEt, 9:1), giving **8g** (0.102 g, 0.325 mmol, 65%), as slightly yellow oil. IR (film) ν/cm^−1^ 744, 790, 1119, 1437, 1719, 2828, 2932; ^1^H-NMR (300 MHz, CDCl_3_) δ 1.78–2.14 (m, 2H), 2.38–2.48 (m, 1H), 2.99–3.14 (m, 1H), 3.21 (s, 3H), 3.24–3.30 (m, 2H), 3.35 (s, 3H), 3.93 (d, 1H, *J =* 9.6), 4.12 (d, 1H, *J =* 9.9), 7.11 (t, 1H, *J =* 7.8), 7.31 (dd, 1H, *J =* 1.2, 7.8), 7.57 (dd, 1H, *J =* 1.2, 7.8);^13^C-NMR (75 MHz, CDCl_3_) δ 26.0, 30.1, 38.6, 51.6, 59.7, 72.3, 86.5, 125.9, 126.9, 127.7, 133.4, 137.7, 139.4, 209.1. LRMS *m/z* (%) 45 (62), 89 (18), 115 (34), 128 (100), 209 (16), 220 (17), 239/241 (33), 267/269 (21), 312/314 (M^+•^, 5); HRMS (ESI) *m/z*, calcd for [C_14_H_17_79BrO_3_+Na]^+^: 335.0259, found: 335.0261, calcd for [C_14_H_17_81BrO_3_+Na]^+^: 337.0238, found: 337.0230.

*5-Methoxy-5-(methoxymethyl)-1-tosyl-1,2,3,5-tetrahydro-4H-benzo[b]azepin-4-one* (**8l**). The reaction was performed as described for **8g**, using a solution of **3l** (0.197 g, 0.490 mmol) and of PTSA (0.020 g, 0.12 mmol, 24 mol %) in MeOH (3 mL), and HTIB (0.481 g, 1.23 mmol). The residue was purified by flash column chromatography (hexanos/AcOEt/CH_2_Cl_2_, 5:1:4), giving **8l** (0.135 g, 0.346 mmol, 69%), as slightly yellow solid.mp 100.4–100.7 °C; IR (film) ν/cm^−1^ 1159, 1348, 1454, 1487, 1720, 2827, 2930; ^1^H-NMR (300 MHz, CDCl_3_) δ 2.45 (s, 3H), 2.51–2.58 (m, 1H), 3.06–3.14 (m, 1H), 3.23 (s, 3H), 3.31 (s, 3H), 3.73 (d, 1H, *J =* 10.8), 3.83 (bs, 2H), 4.19 (d, 1H, *J =* 10.5), 7.29–7.38 (m, 5H), 7.55–7.57 (m, 1H), 7.77 (dt, 2H, *J =* 1.8, 3.9, 8.4); ^13^C-NMR (75 MHz, CDCl_3_) δ 21.6, 38.8, 48.6, 52.5, 59.6, 72.5, 85.4, 127.5, 127.7 (2C), 128.1, 129.3, 129.5, 129.8 (2C), 137.3 (2C), 144.0 (2C), 205.4; LRMS *m/z* (%) 45 (100), 65 (52), 77 (27), 91 (87), 130 (33), 389 (M^+•^, 1); HRMS (ESI) *m/z*, calcd for [C_20_H_23_NO_5_S+Na]^+^: 412.1195, found: 412.1182.

The compounds **5f** + **8f** (1:1), **8d**, **8g**, **12**, **15** and **22l **were evaluated* in vitro* against a panel of nine cell lines [U251 (glioma); UACC-62 (melanoma); MCF-7 (breast); NCI-ADR/RES (ovarian resistant to multiple drugs); 786–0 (kidney); NCI-H460 (lung, non small cells); PC-3 (prostate); HT29 (colon); K562 (leukemia)] kindly provided by Frederick MA (National Cancer Institute, Bethesda, MD, USA) and the immortalized human keratinocytes (HaCat) cell line kindly provided by Prof. Dr. Ricardo Della Coletta (University of Campinas, UNICAMP, Campinas, Brazil). Stock and experimental cultures were grown in medium containing 5 mL RPMI 1640 (GIBCO BRL) supplemented with 5% fetal bovine serum (GIBCO BRL). Penicilin/Streptomicin mixture (1000 Um·L^−1^:1000 μg·mL^−1^, 1 mL L^−1^ RPMI) was added to the experimental cultures. Cells in 96-well plates (100 μL cells well^−1^) were exposed to sample concentrations in DMSO/RPMI (0.25, 2.5, 25, 250 μg·mL^−1^) in triplicate at 37 °C, 5% of CO_2_ in air for 48 h. The final DMSO concentration did not affect cell viability. Doxorubicin (0.025 to 25 µg·mL^−1^) was used as positive control. Before (T_0_ plate) and after the sample addition (T_1_ plates), cells were fixed with 50% trichloroacetic acid, and cell proliferation was determined by spectrophotometric quantification (540 nm) of cellular protein using the sulforhodamine B assay. Using the dose-response curve for each cell line, the concentration that totally inhibits cell growth (TGI, expressed in µM) was determined through non-linear regression analysis using ORIGIN software version 8.0 (OriginLab Corporation, Northampton, MA, USA, 2007) [63,70].

## 4. Conclusions

In conclusion, a metal-free approach for the synthesis of seven- and eight- membered rings through an iodine(III)-mediated ring expansion reaction was described. The substrates can be easily obtained from readily available starting materials. The amount of the oxidizer and the reaction conditions can be managed to obtain different products. Moreover, a short route to the synthesis of medium-ring lactones was developed. The antiproliferative activity of new seven-membered ring compounds was evaluated, and the results showed compound **12f** as having a moderated citostatic effect. The results herein described have great potential for application in the chemical synthesis of seven-membered rings.

## Supplementary Materials

Supplementary materials can be accessed at: http://www.mdpi.com/1420-3049/20/01/1475/s1.
