# Supplementary material for: Seven-Membered Rings through Metal-Free Rearrangement Mediated by Hypervalent Iodine
Source: Molecules. 2015 Jan 15;20(1):1475–94. doi: 10.3390/molecules20011475 (PMC6272645; doi:10.3390/molecules20011475)
Supplement: Supplementary file 1 [file molecules-20-01475-s001.pdf]

Supplementary Materials

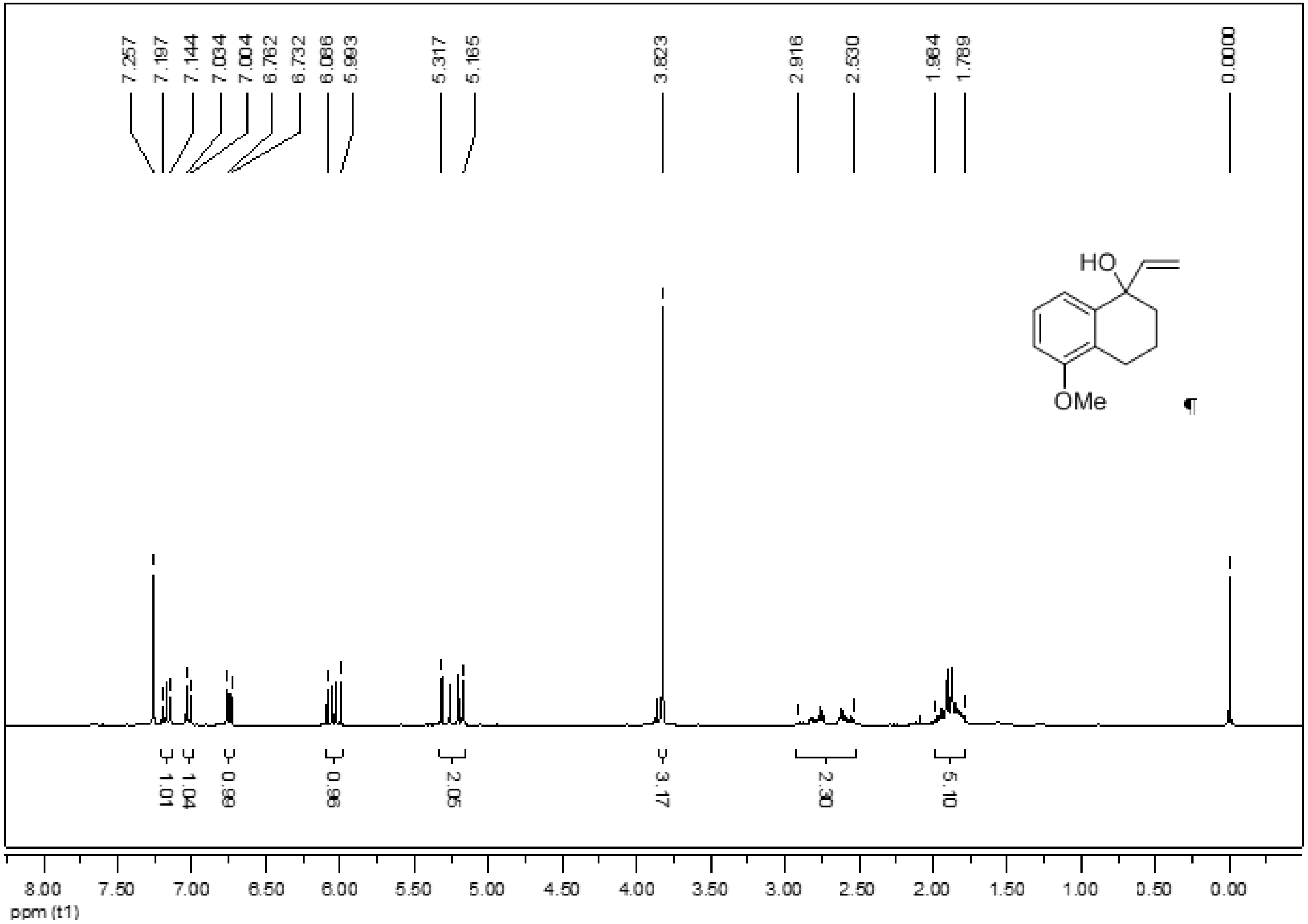

Figure S1. ^{1}H-NMR spectra of 5-methoxy-1-vinyl-1,2,3,4-tetrahydronaphthalen-1-ol (**2d**) (300 MHz, $CDCl_3$).

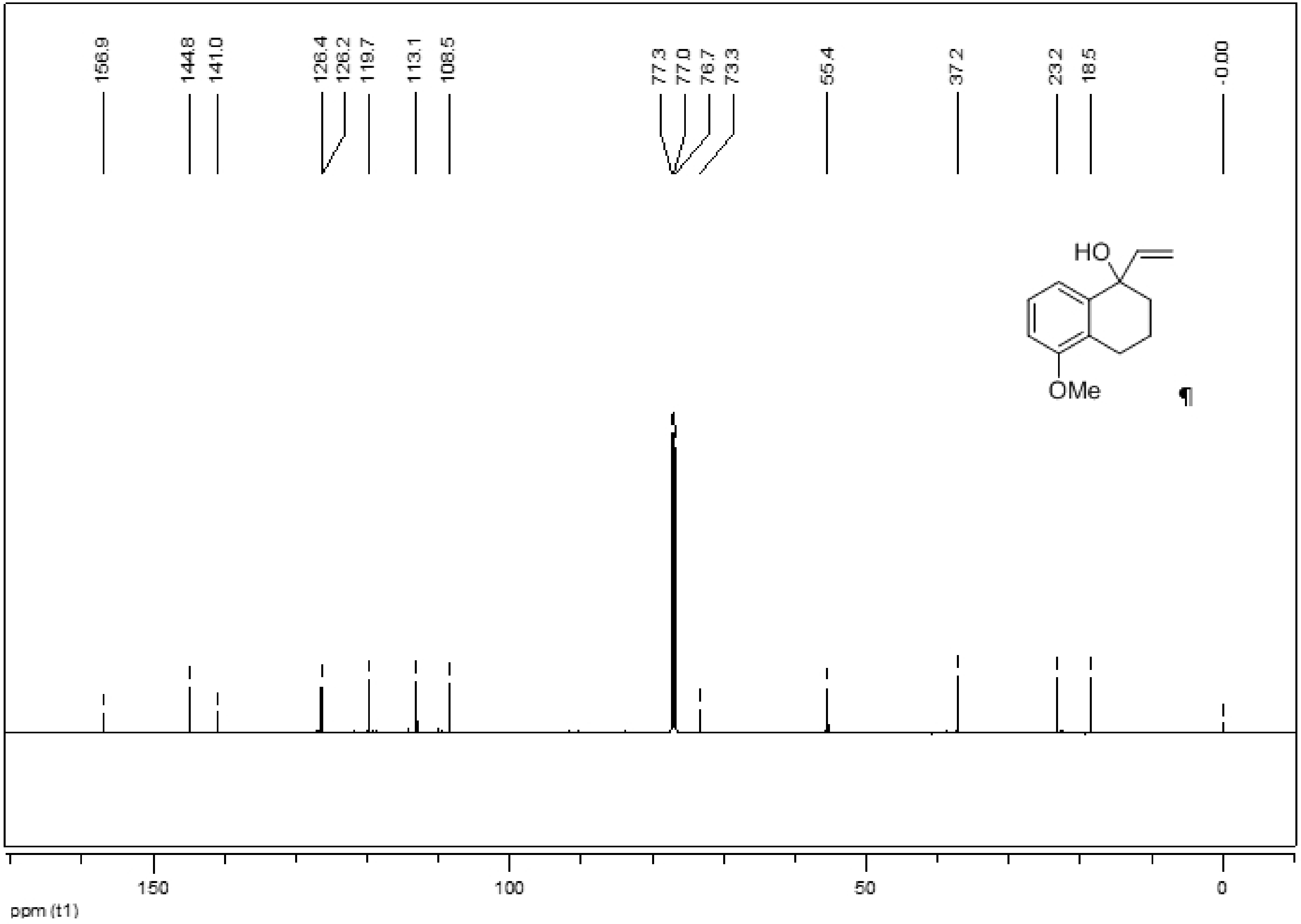

Figure S2. ^{13}C-NMR spectra of 5-methoxy-1-vinyl-1,2,3,4-tetrahydronaphthalen-1-ol (**2d**) (75 MHz, $CDCl_3$).

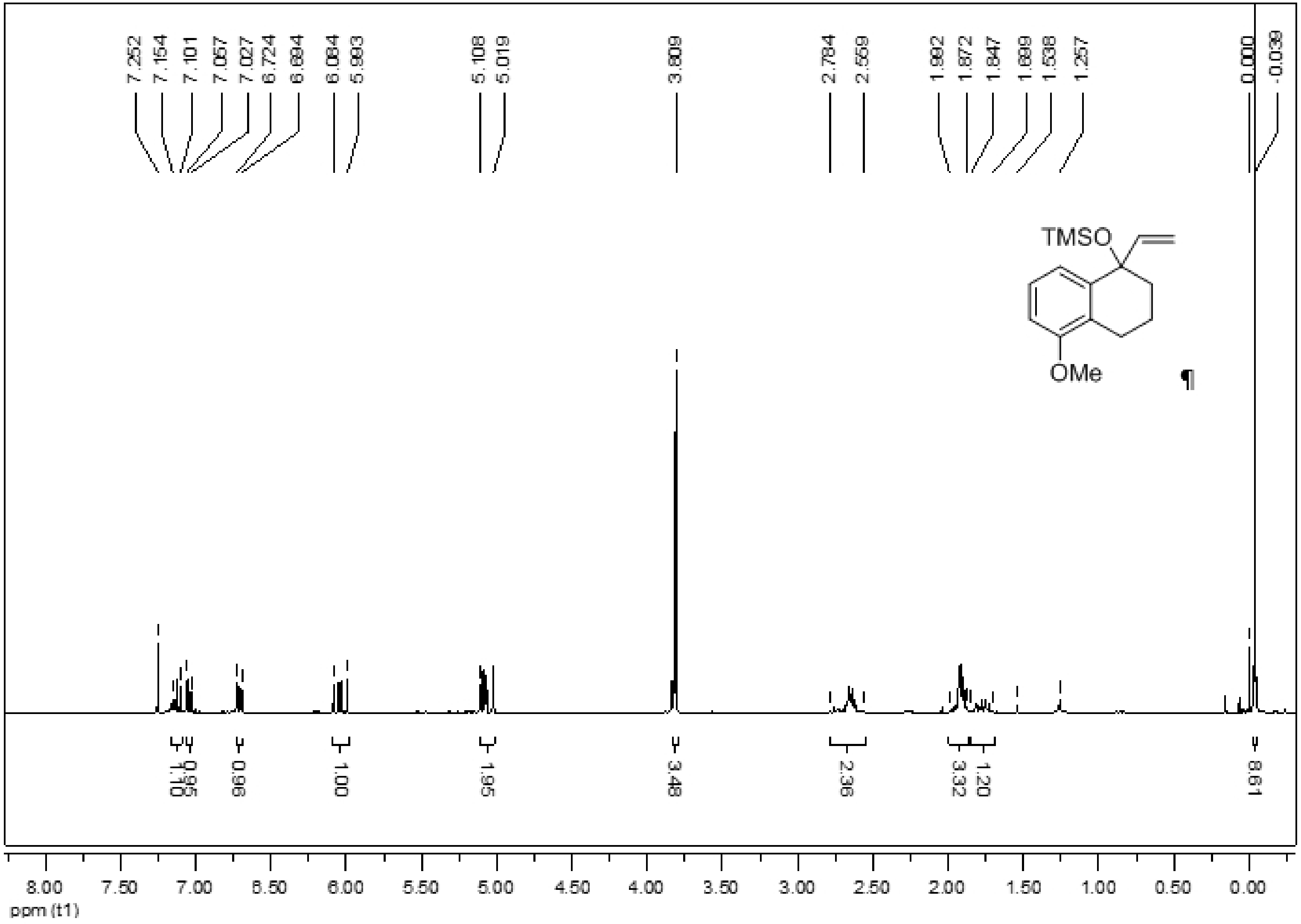

Figure S3. ^{1}H-NMR spectra of ((5-methoxy-1-vinyl-1,2,3,4-tetrahydronaphthalen-1-yl)oxy)trimethylsilane (**3d**) (300 MHz, $CDCl_3$).

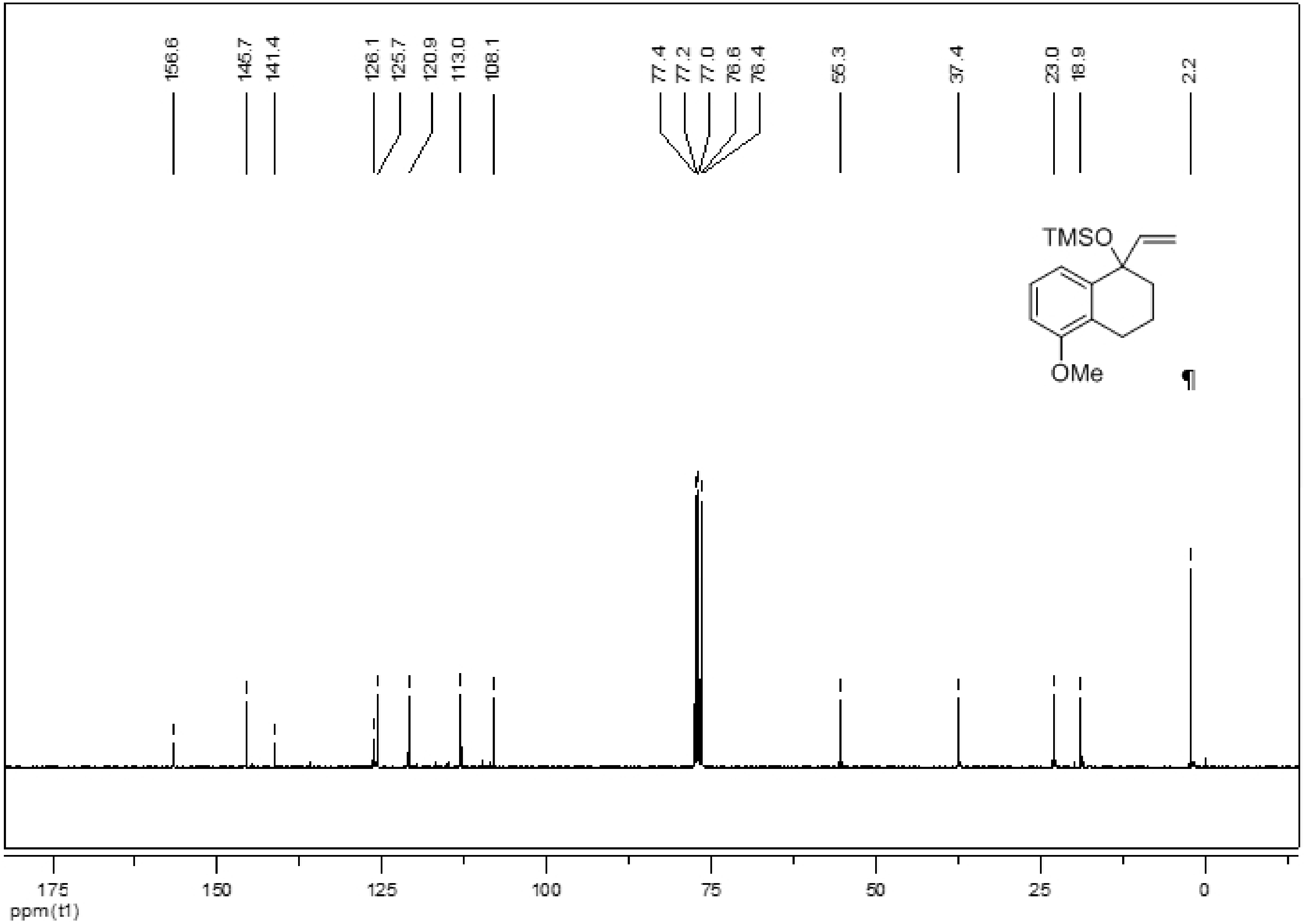

Figure S4. ^{13}C-NMR spectra of ((5-methoxy-1-vinyl-1,2,3,4-tetrahydronaphthalen-1-yl)oxy)trimethylsilane (**3d**) (75 MHz, $CDCl_3$).

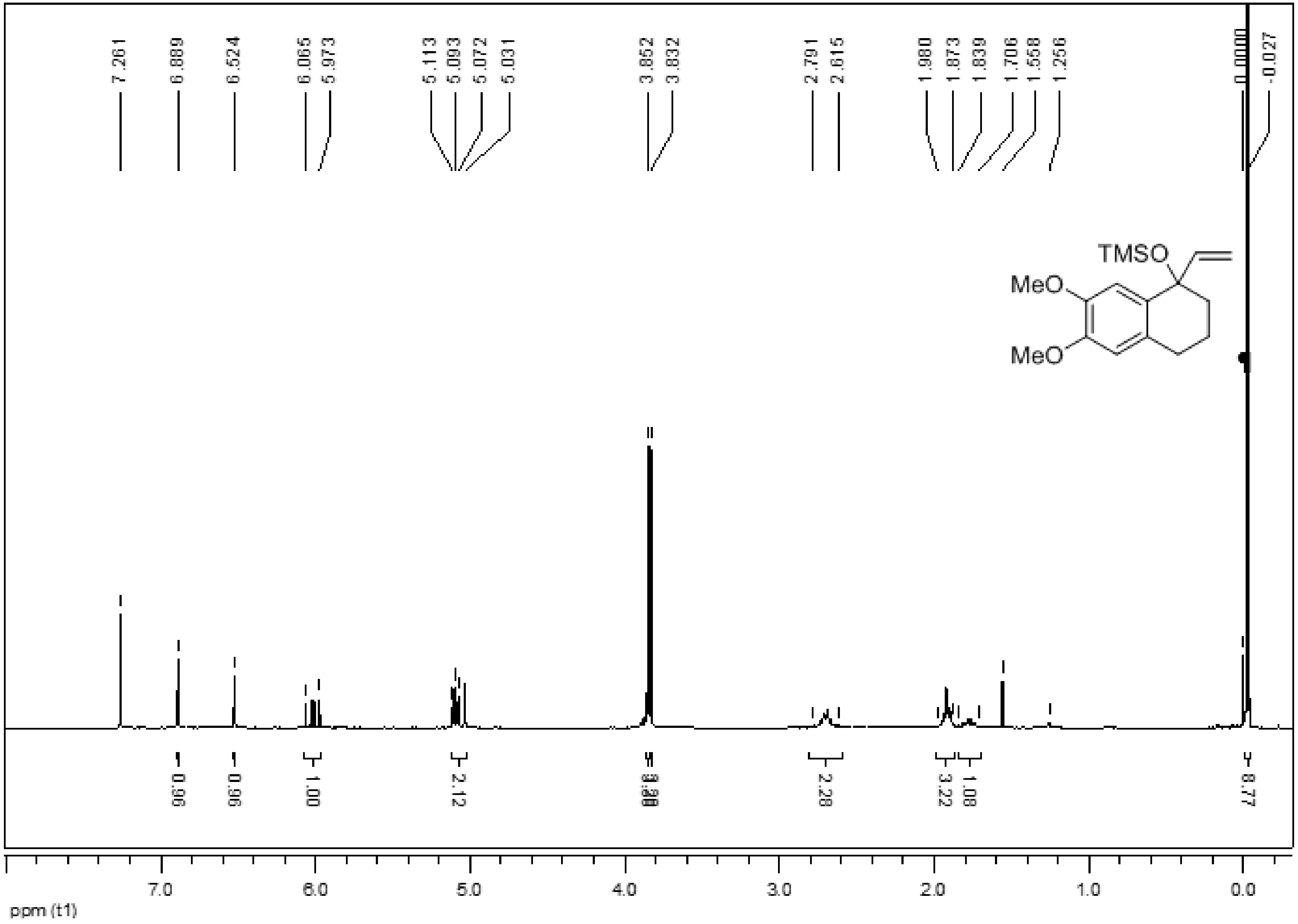

Figure S5. ^{1}H-NMR spectra of ((6,7-dimethoxy-1-vinyl-1,2,3,4-tetrahydronaphthalen-1-yl)oxy)trimethylsilane (**3f**) (300 MHz, $CDCl_3$).

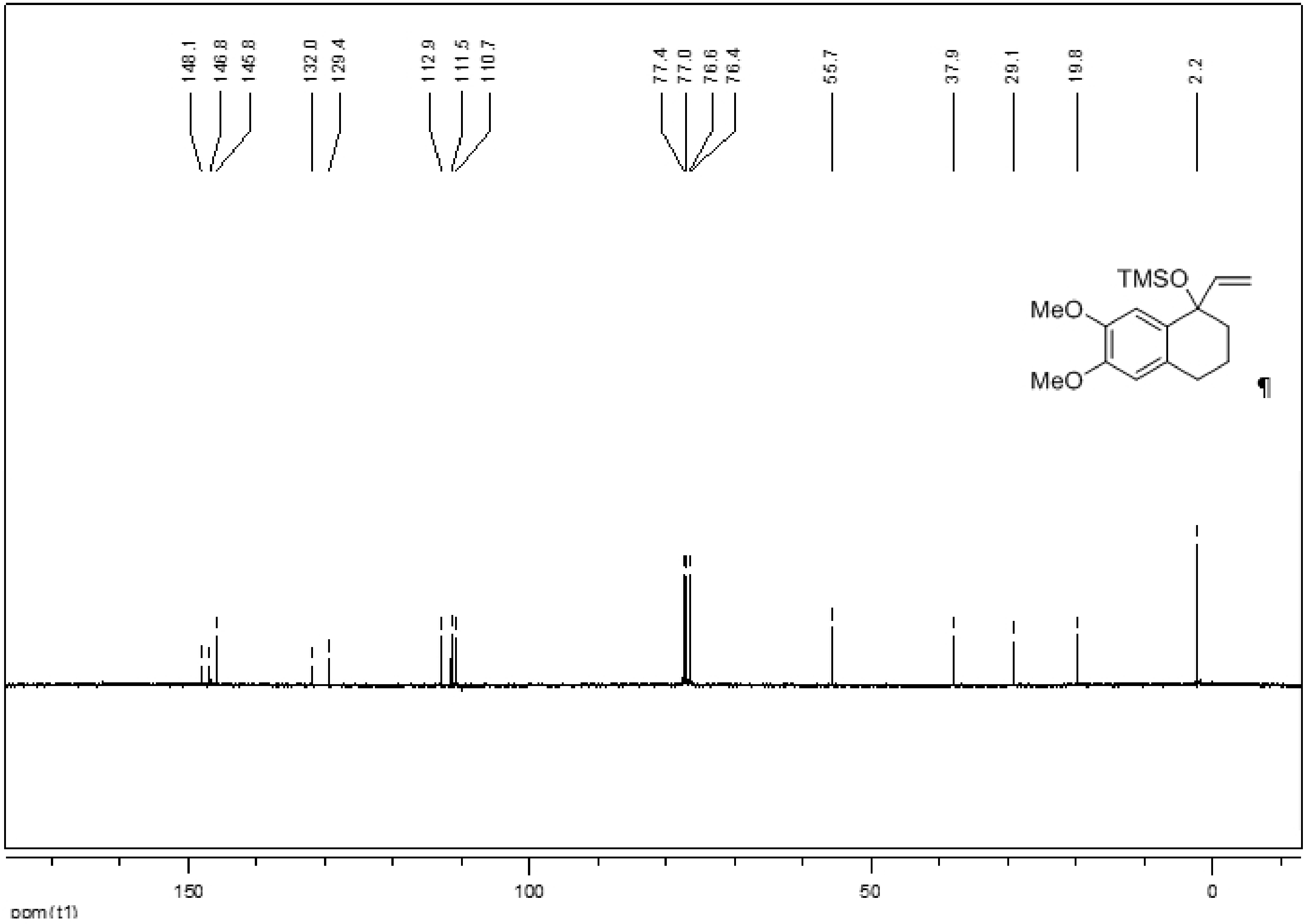

Figure S6. ^{13}C-NMR spectra of ((6,7-dimethoxy-1-vinyl-1,2,3,4-tetrahydronaphthalen-1-yl)oxy)trimethylsilane (**3f**) (75 MHz, $CDCl_3$).

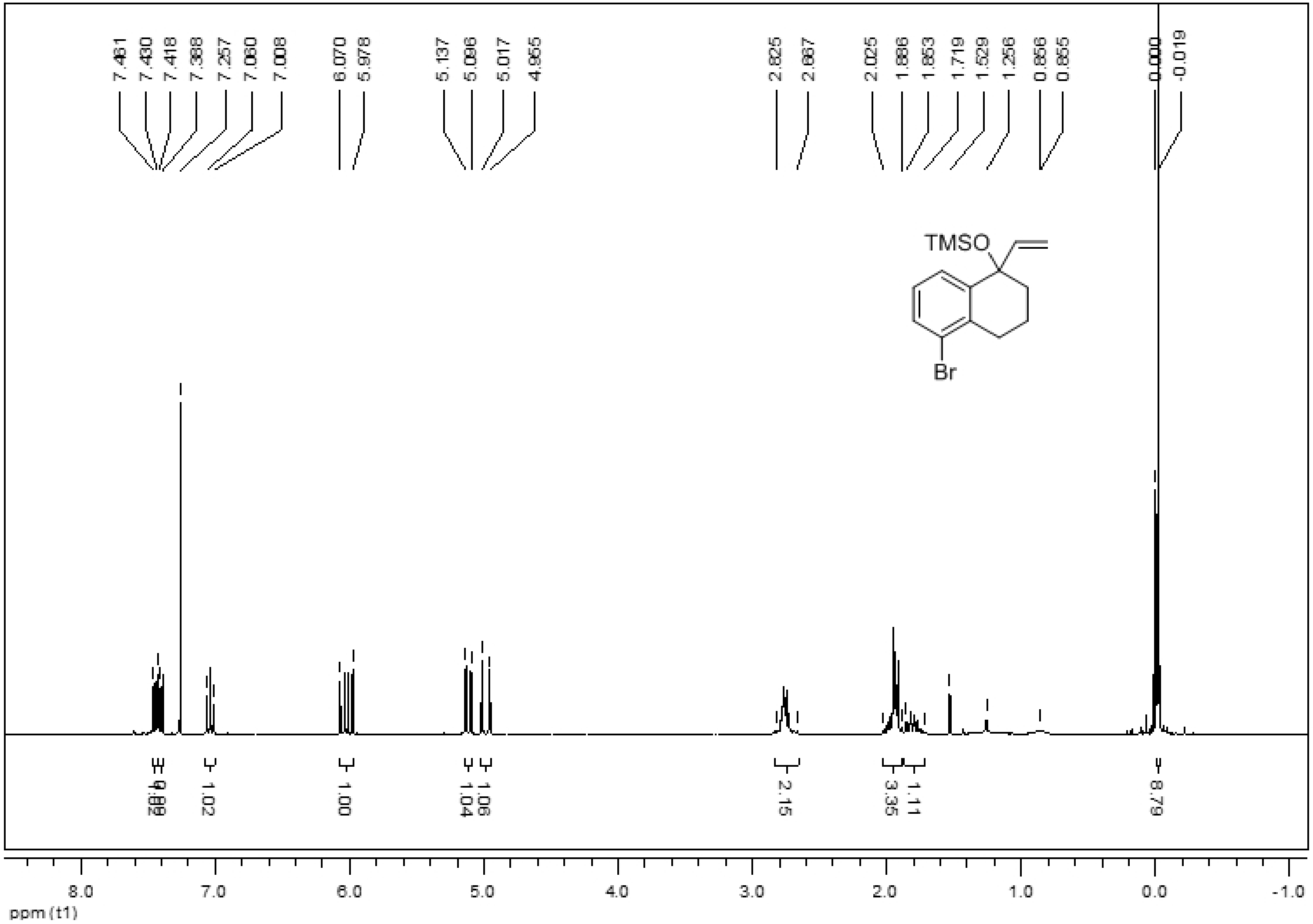

Figure S7. ^{1}H-NMR spectra of ((5-bromo-1-vinyl-1,2,3,4-tetrahydronaphthalen-1-yl)oxy)trimethylsilane (**3g**) (300 MHz, $CDCl_3$).

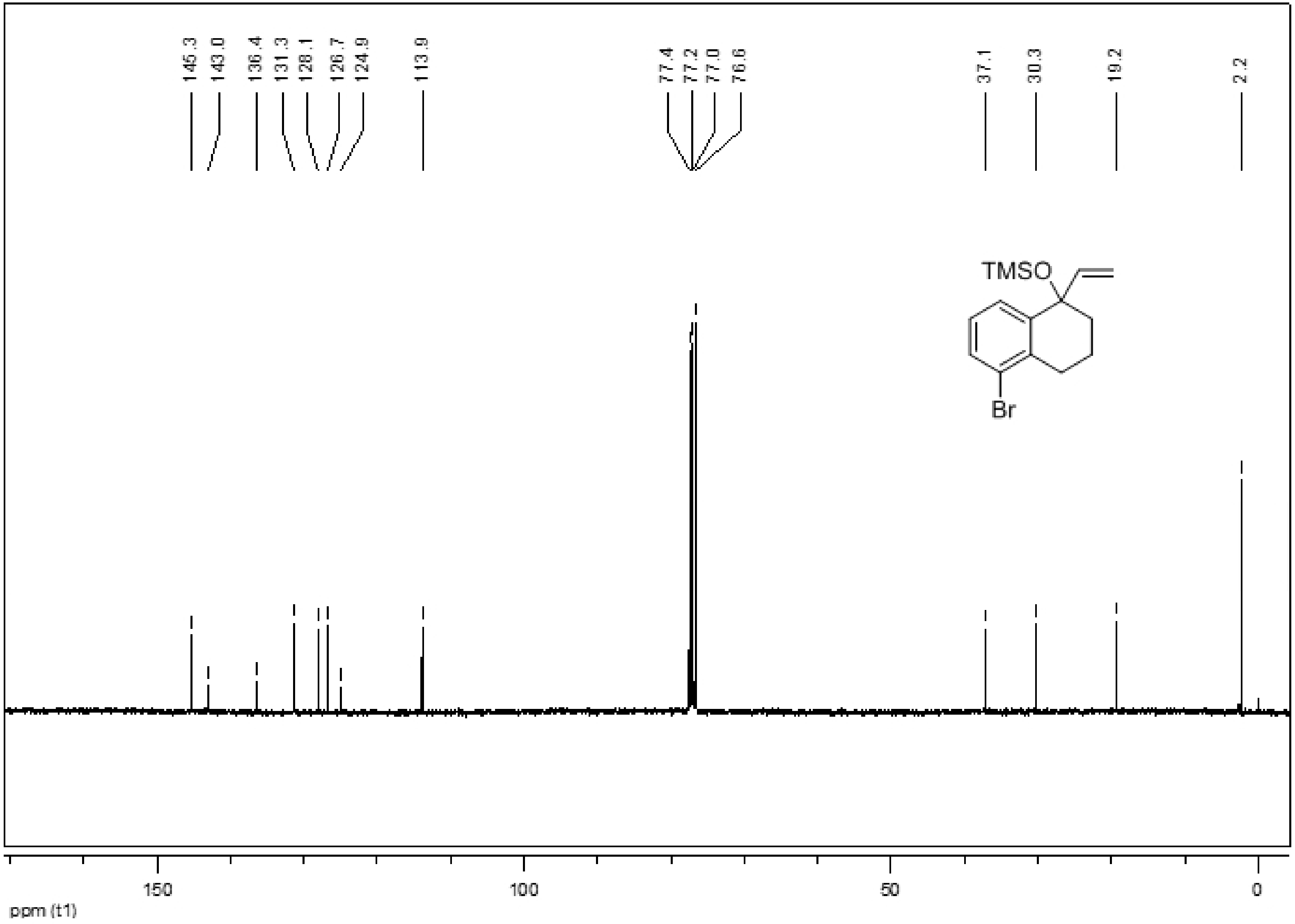

Figure S8. ^{13}C-NMR spectra of ((5-bromo-1-vinyl-1,2,3,4-tetrahydronaphthalen-1-yl)oxy)trimethylsilane (**3g**) (75 MHz, $CDCl_3$).

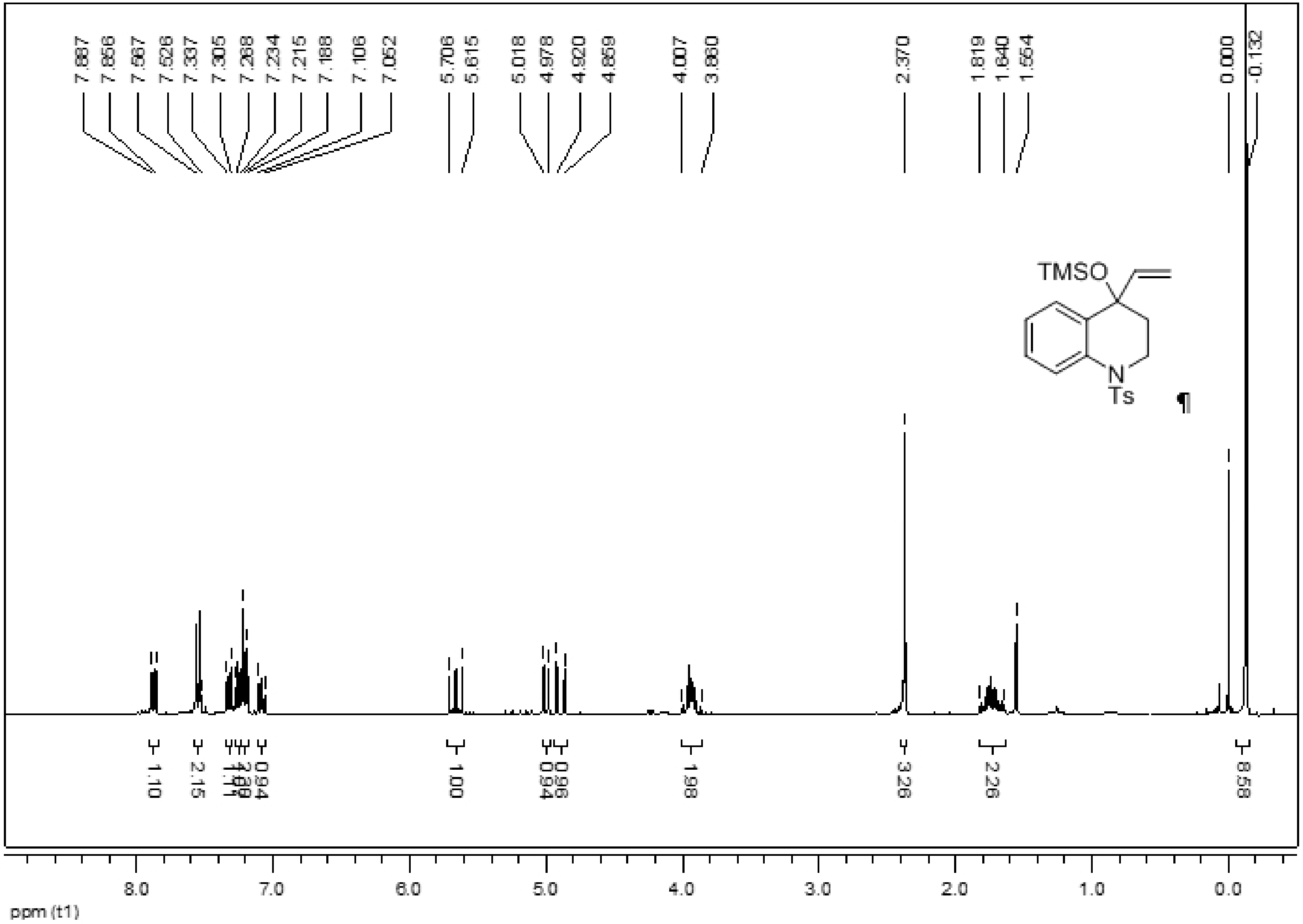

Figure S9. ^{1}H-NMR spectra of 1-tosyl-4-((trimethylsilyl)oxy)-4-vinyl-1,2,3,4-tetrahydroquinoline (**3l**) (300 MHz, $CDCl_3$).

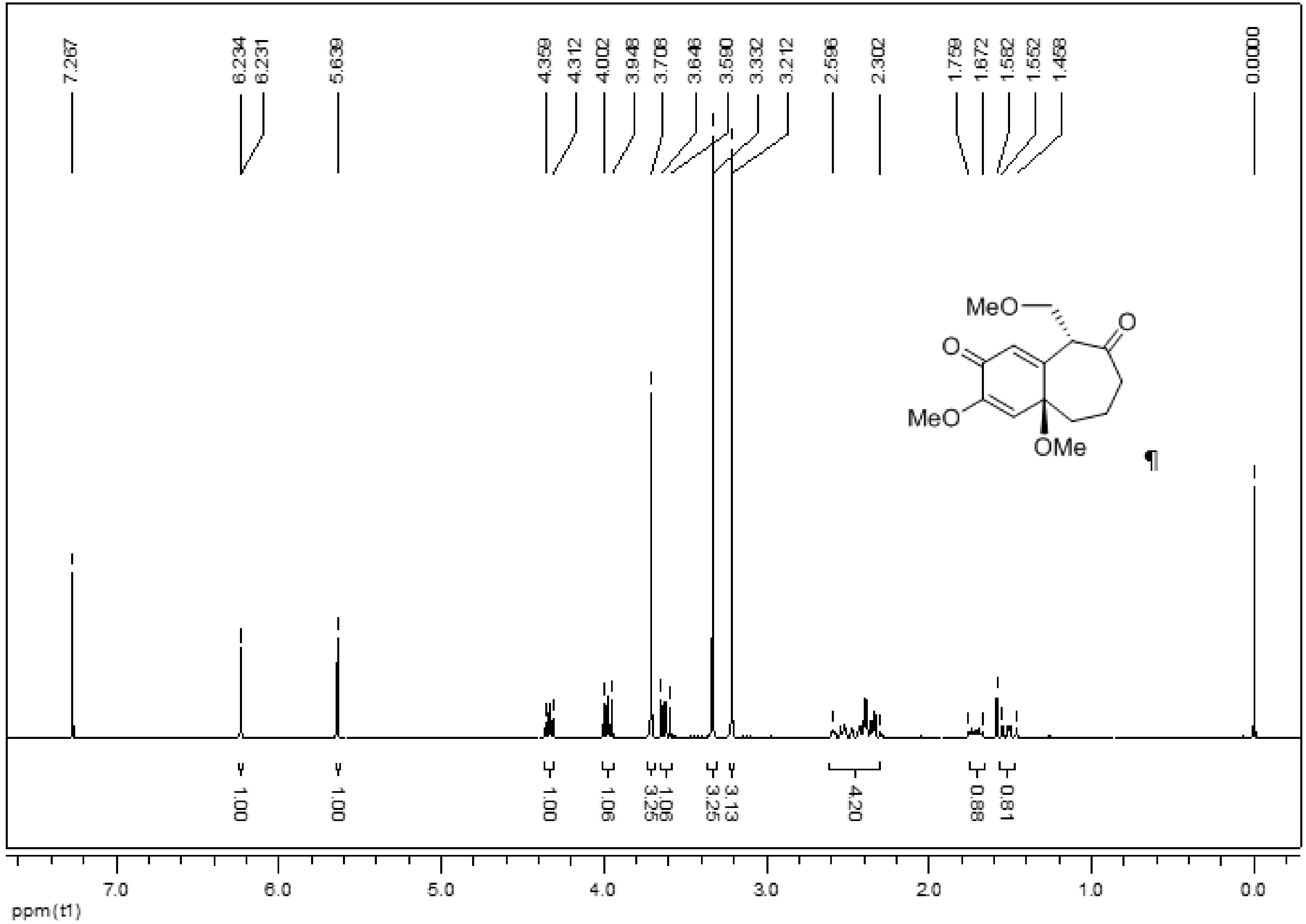

Figure S10. ^{13}C-NMR spectra of 1-tosyl-4-((trimethylsilyl)oxy)-4-vinyl-1,2,3,4-tetrahydroquinoline (**3l**) (75 MHz, $CDCl_3$).

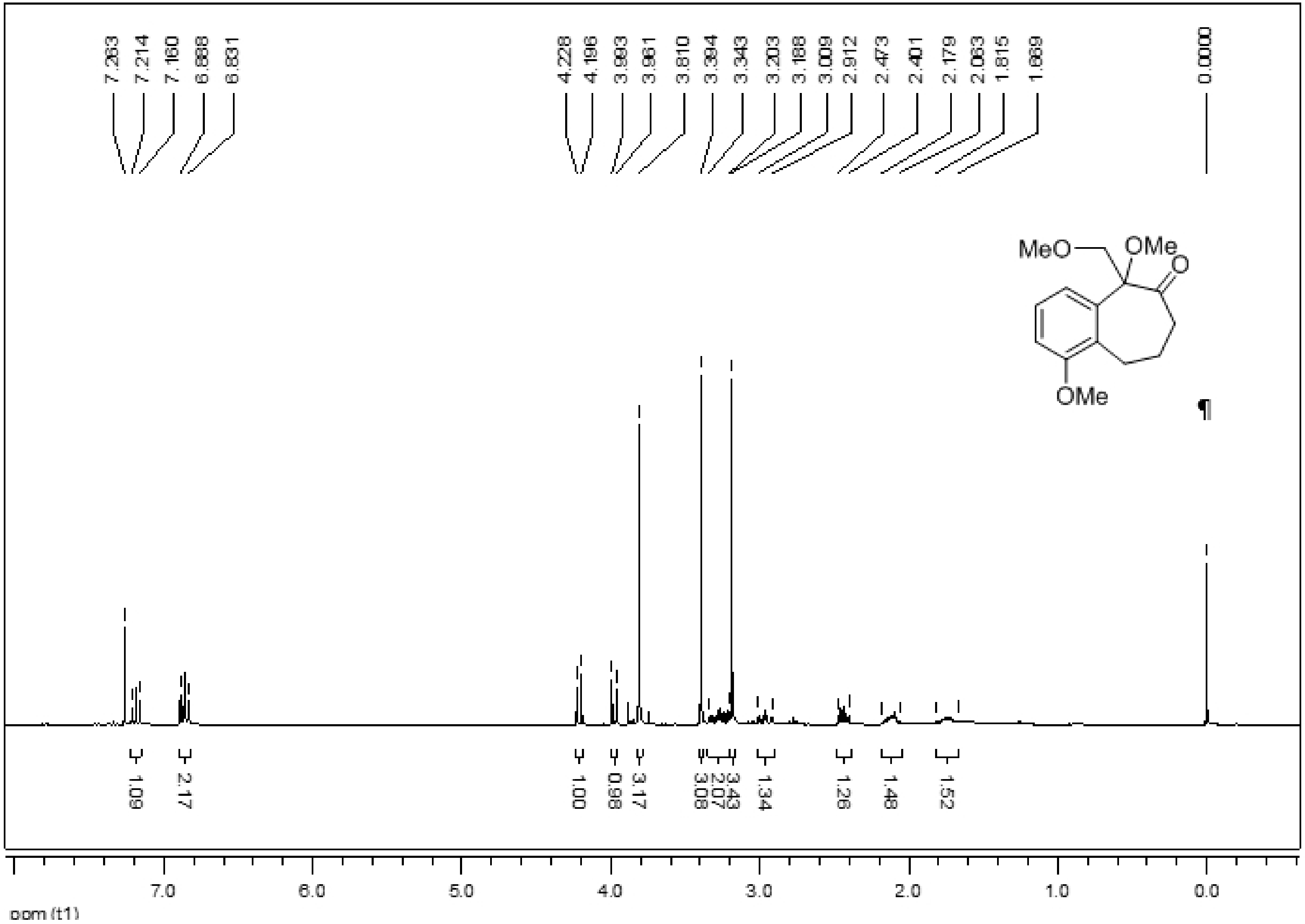

Figure S11. ^{1}H-NMR spectra of 1,5-dimethoxy-5-(methoxymethyl)-5,7,8,9-tetrahydro-6*H*-benzo[7]annulen-6-one (**8d**) (300 MHz, $CDCl_3$).

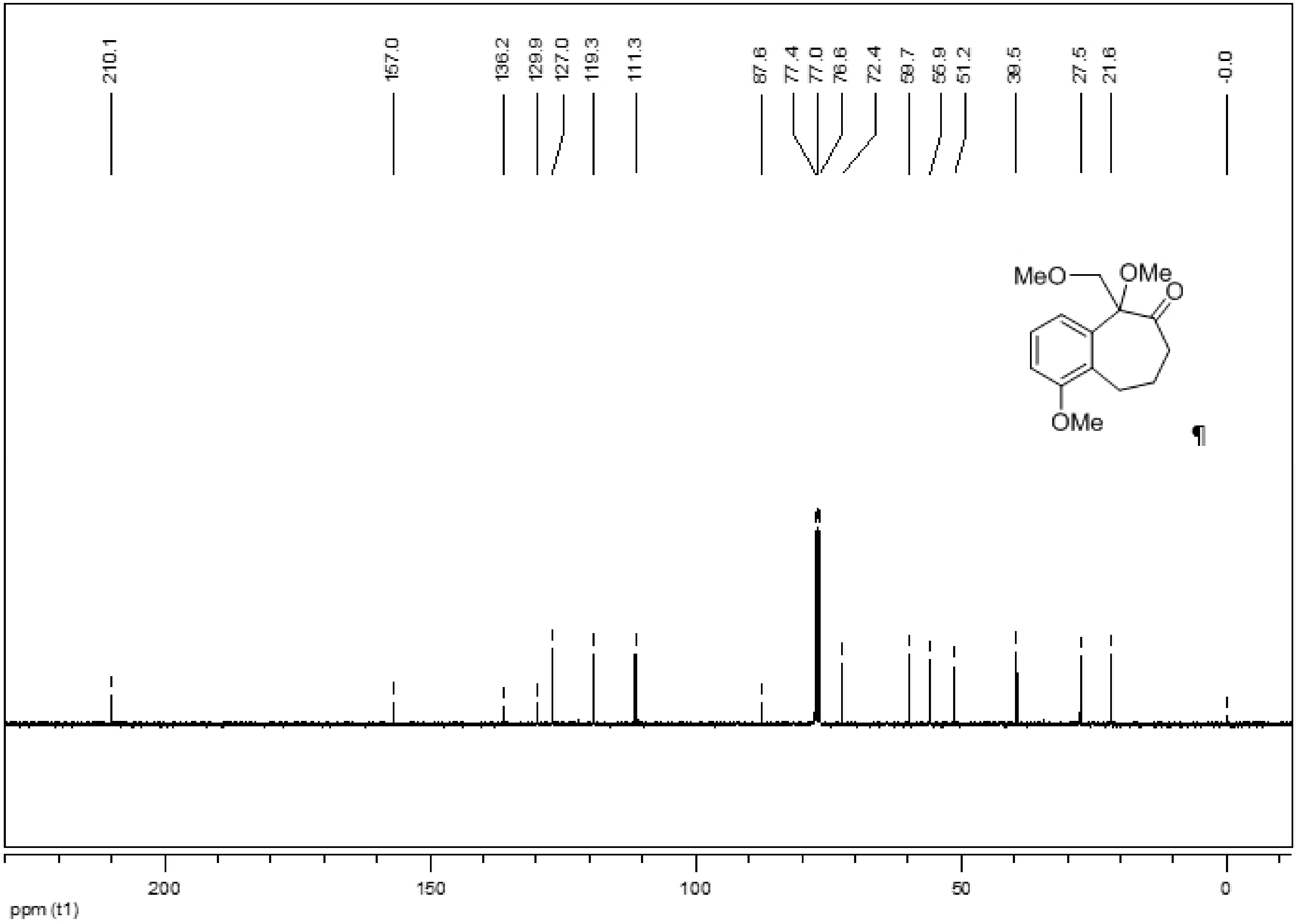

Figure S12. ^{13}C-NMR spectra of 1,5-dimethoxy-5-(methoxymethyl)-5,7,8,9-tetrahydro-6*H*-benzo[7]annulen-6-one (**8d**) (75 MHz, $CDCl_3$).

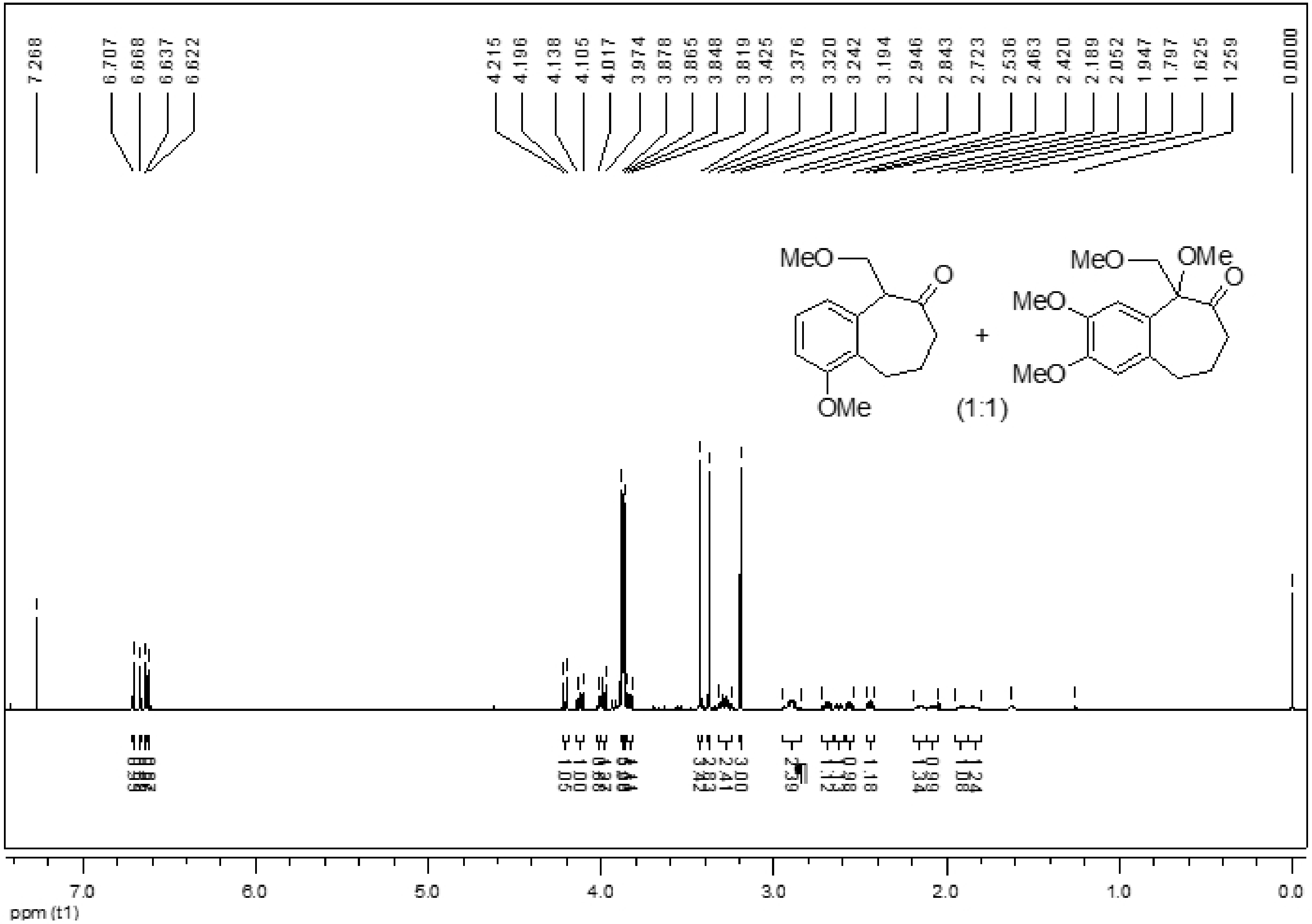

Figure S13. ^{1}H-NMR spectra of 2,3-dimethoxy-5-(methoxymethyl)-5,7,8,9-tetrahydro-6*H*-benzo[7]annulen-6-one (5f) and 2,3,5-Trimethoxy-5-(methoxymethyl)-5,7,8,9-tetrahydro-6*H*-benzo[7]annulen-6-one (**8f**) (1:1) (500 MHz, $CDCl_3$).

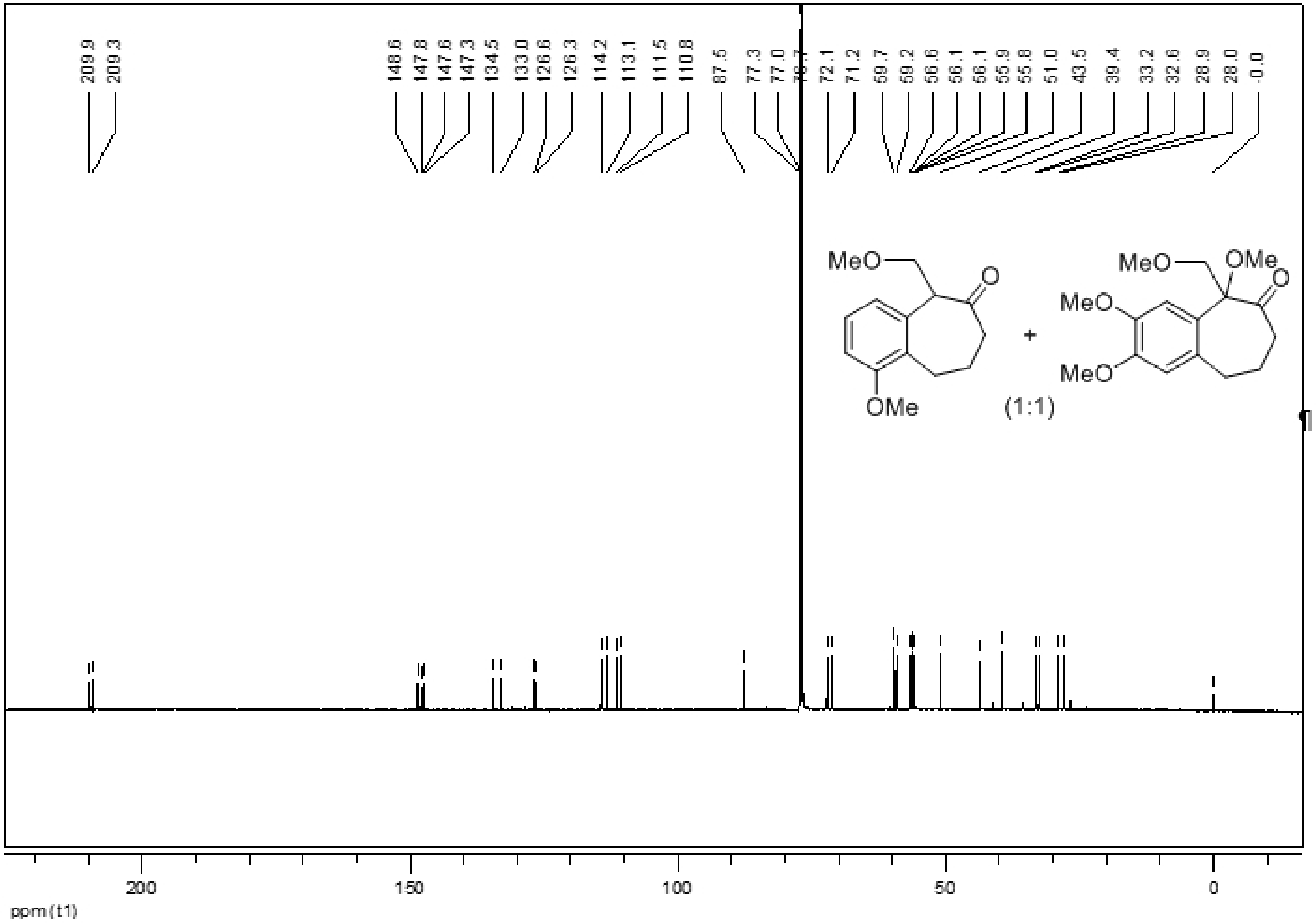

Figure S14. ^{13}C-NMR spectra of 2,3-Dimethoxy-5-(methoxymethyl)-5,7,8,9-tetrahydro-6*H*-benzo[7]annulen-6-one (5f) and 2,3,5-Trimethoxy-5-(methoxymethyl)-5,7,8,9-tetrahydro-6*H*-benzo[7]annulen-6-one (**8f**) (1:1) (125 MHz, $CDCl_3$).

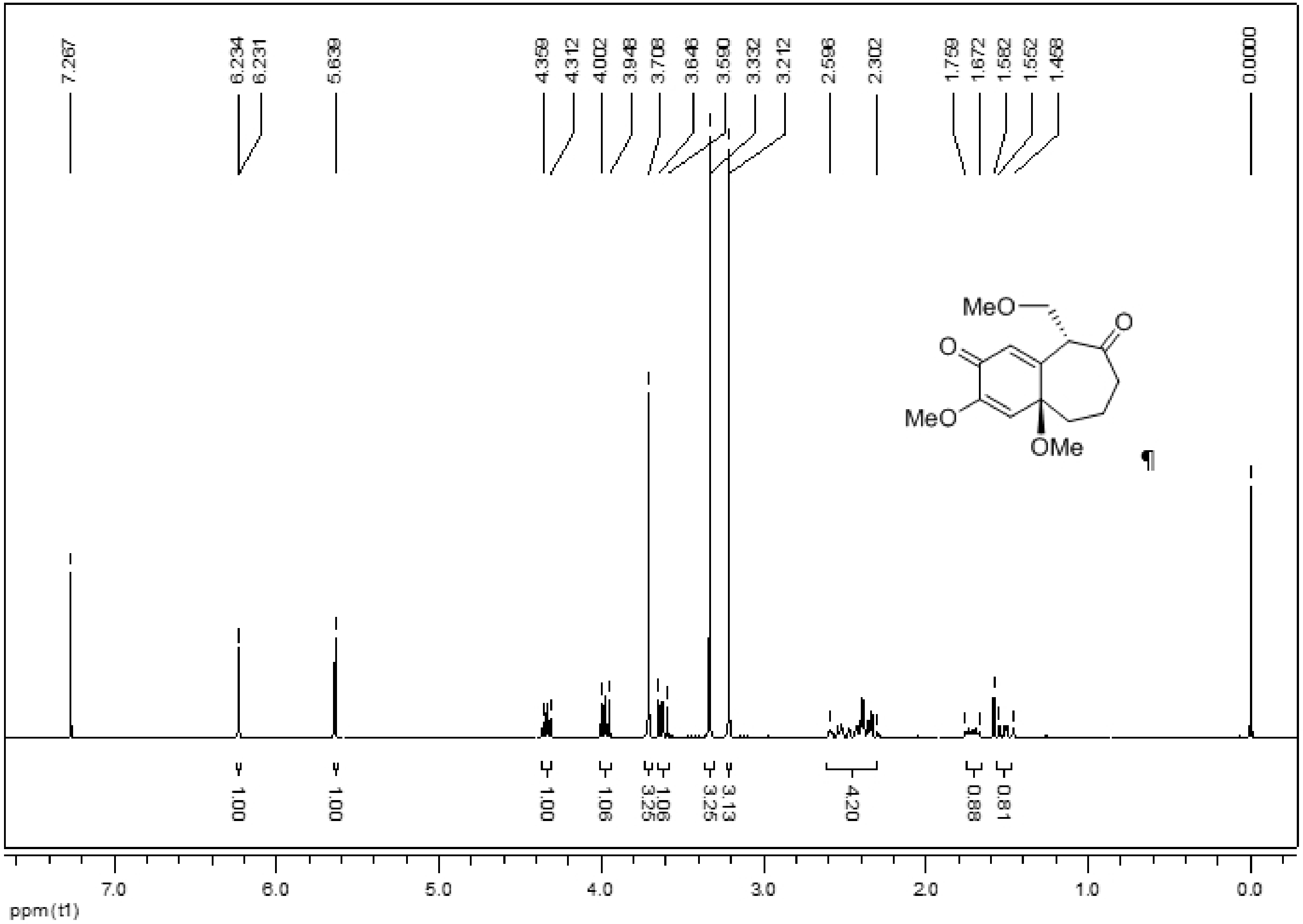

Figure S15. ^{1}H-NMR spectra of *trans*-3,4a-Dimethoxy-9-(methoxymethyl)-4a,5,6,7-tetrahydro-2*H*-benzo[7]annulene-2,8(9*H*)-dione (**12f**) (300 MHz, $CDCl_3$).

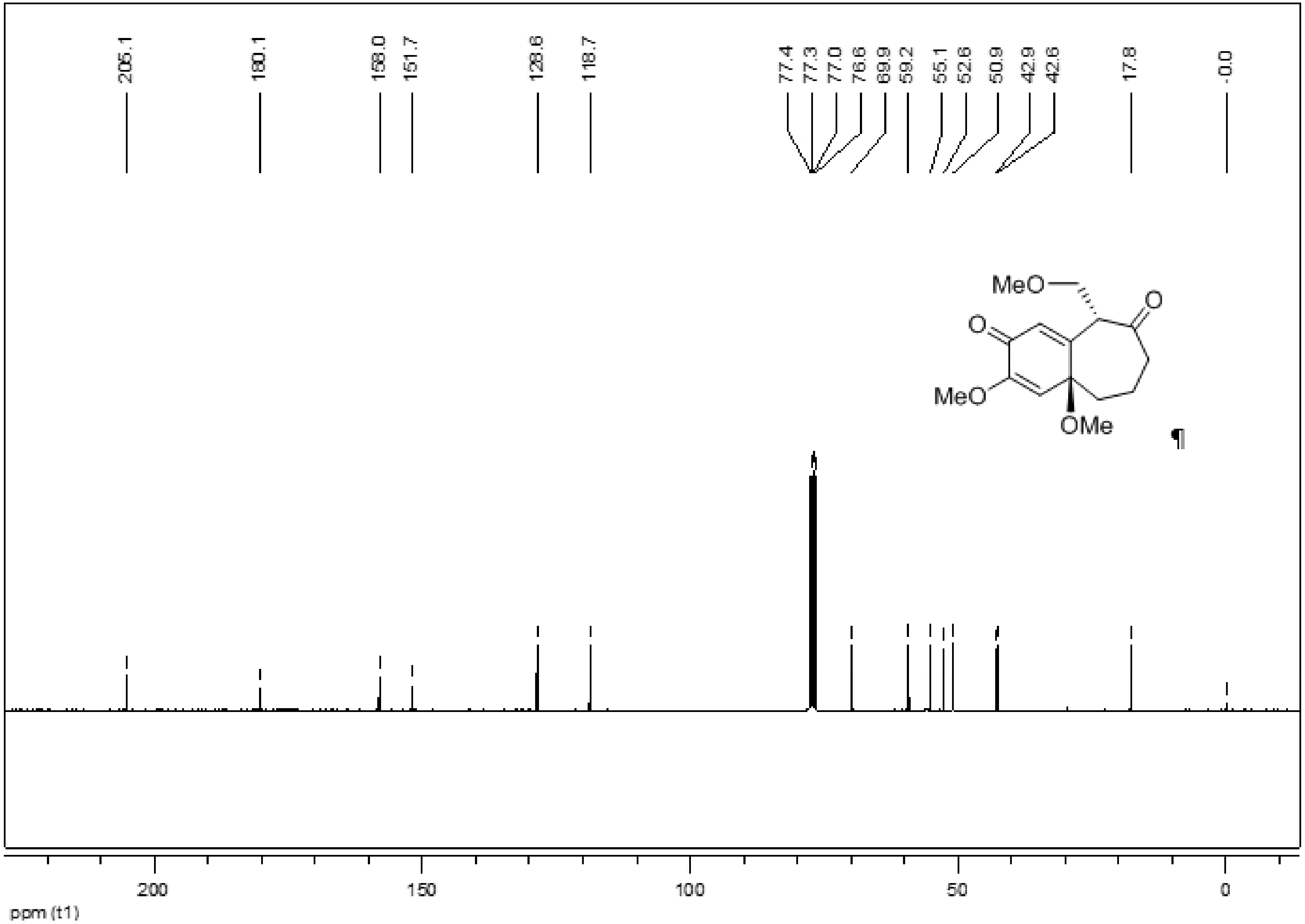

Figure S16. ^{13}C-NMR spectra of *trans*-3,4a-dimethoxy-9-(methoxymethyl)-4a,5,6,7-tetrahydro-2*H*-benzo[7]annulene-2,8(9*H*)-dione (**12f**) (75 MHz, $CDCl_3$).

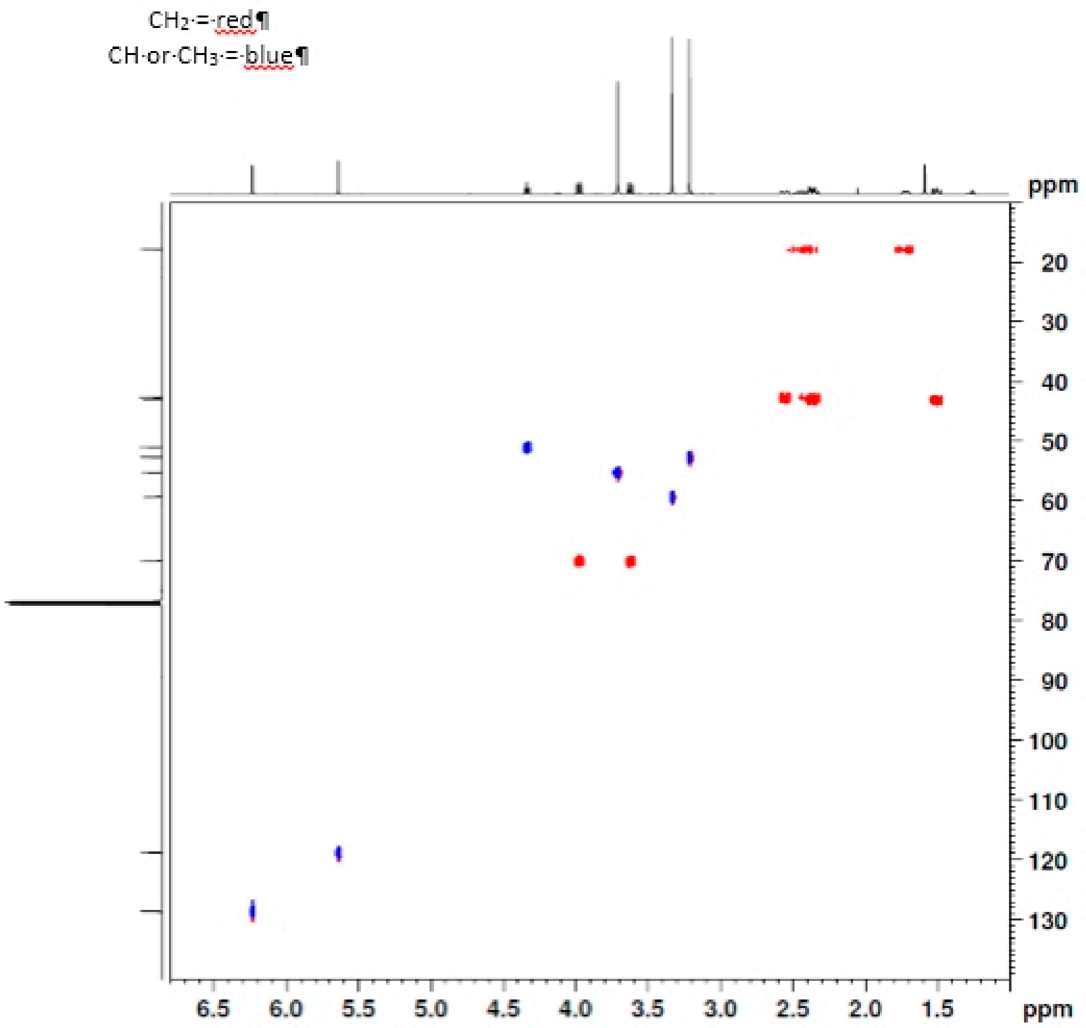

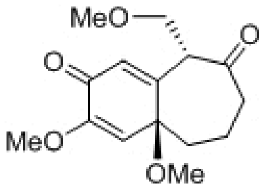

Figure S17. ^{1}H-^{13}C HSQC spectra of *trans*-3,4a-dimethoxy-9-(methoxymethyl)-4a,5,6,7-tetrahydro-2*H*-benzo[7]annulene-2,8(9*H*)-dione (**12f**) (500 MHz, $CDCl_3$.

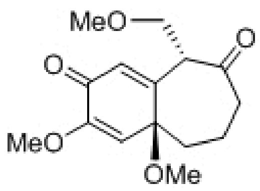

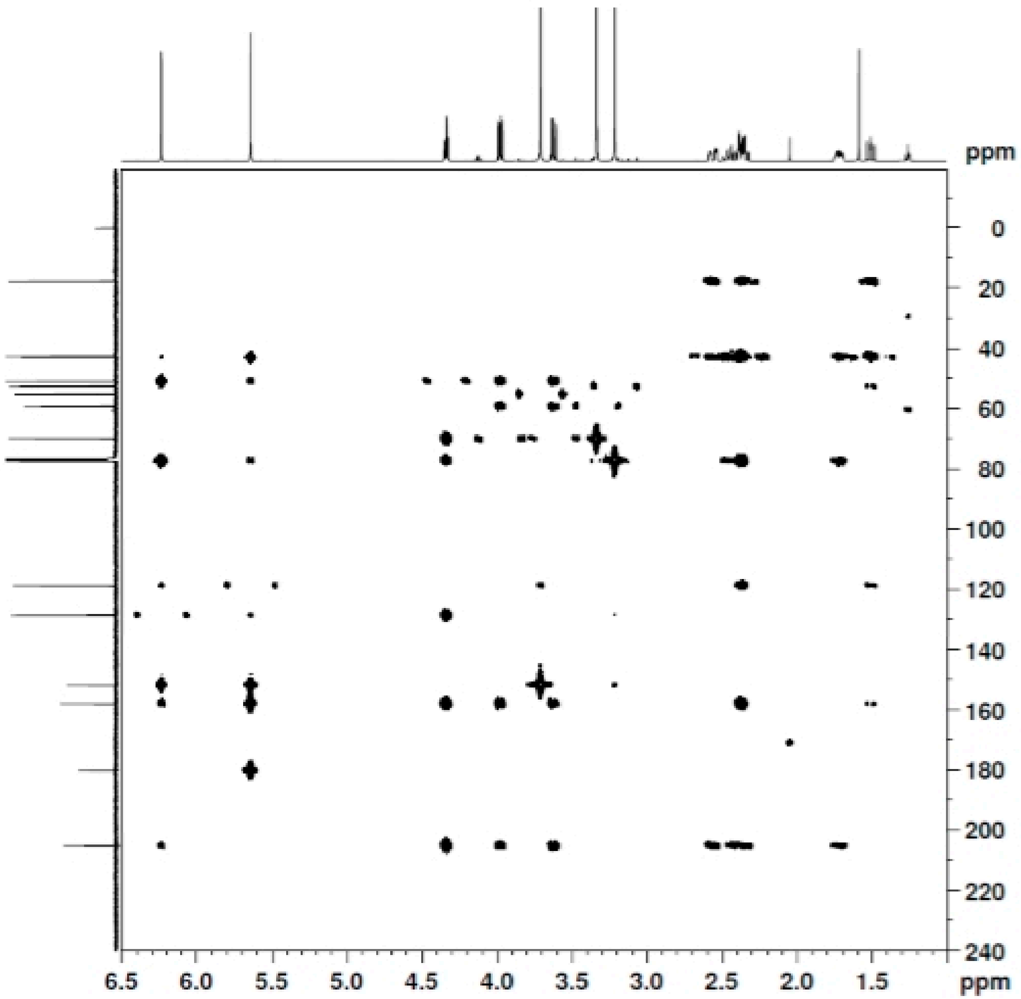

Figure S18. ^{1}H-^{13}C HMBC spectra of *trans*-3,4a-dimethoxy-9-(methoxymethyl)-4a,5,6,7-tetrahydro-2*H*-benzo[7]annulene-2,8(9*H*)-dione (**12f**) (500 MHz, $CDCl_3$).

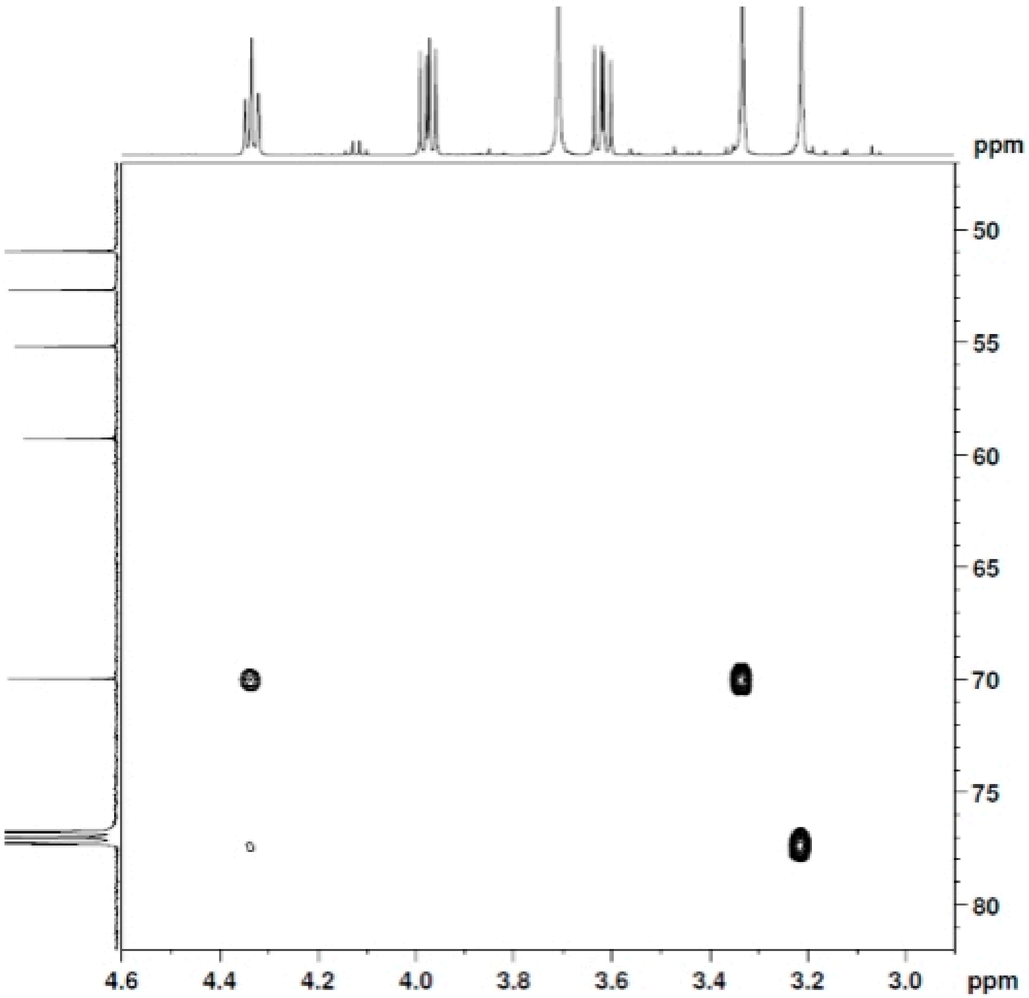

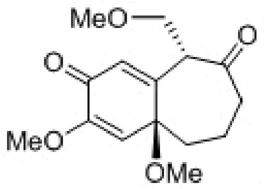

Figure S19. Expansion of ^{1}H-^{13}C HMBC spectra of *trans*-3,4a-dimethoxy-9-(methoxymethyl)-4a,5,6,7-tetrahydro-2*H*-benzo[7]annulene-2,8(9*H*)-dione (**12f**) (500 MHz, $CDCl_3$).

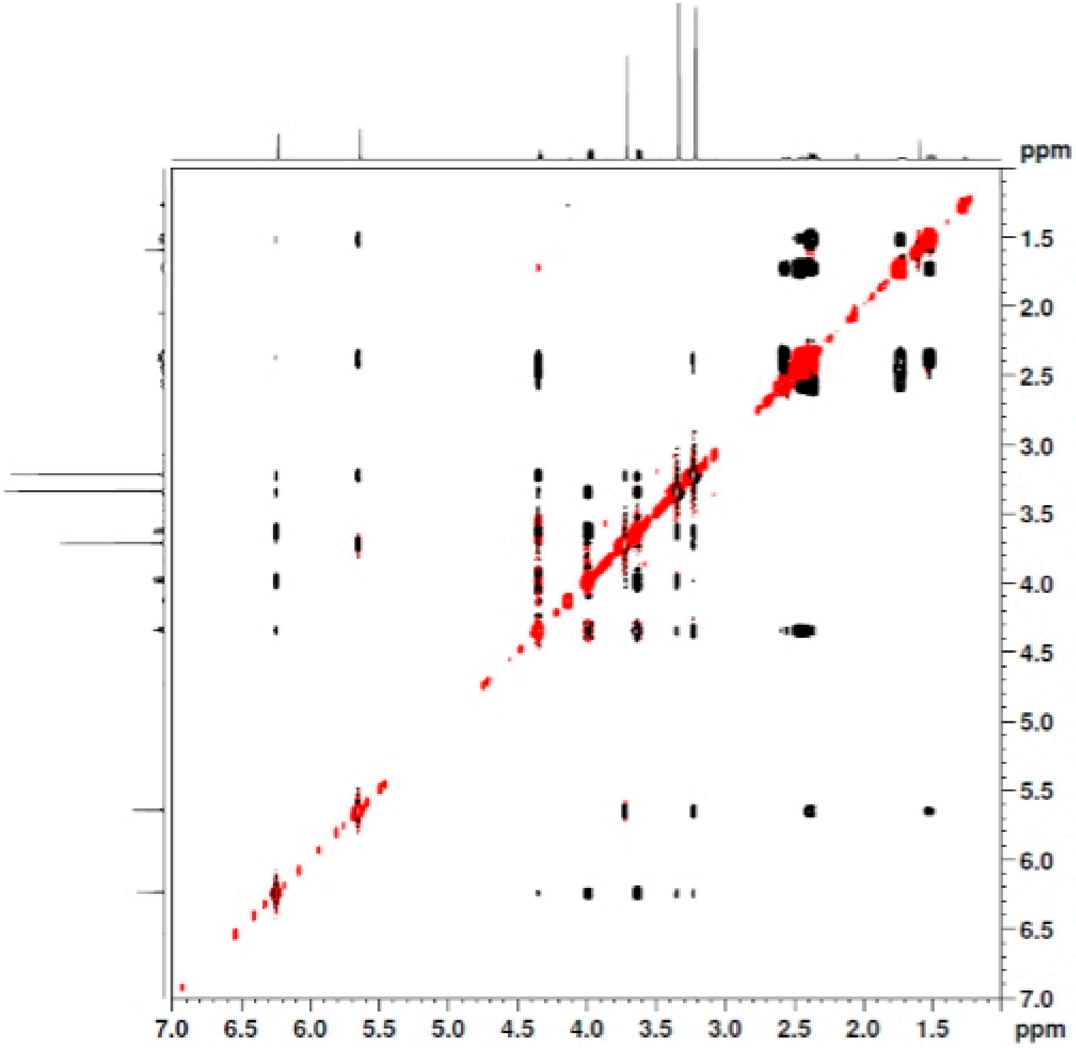

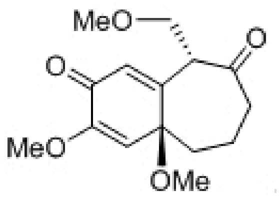

Figure S20. ^{1}H-^{1}H NOESY spectra of *trans*-3,4a-dimethoxy-9-(methoxymethyl)-4a,5,6,7-tetrahydro-2*H*-benzo[7]annulene-2,8(9*H*)-dione (**12f**) (500 MHz, $CDCl_3$).

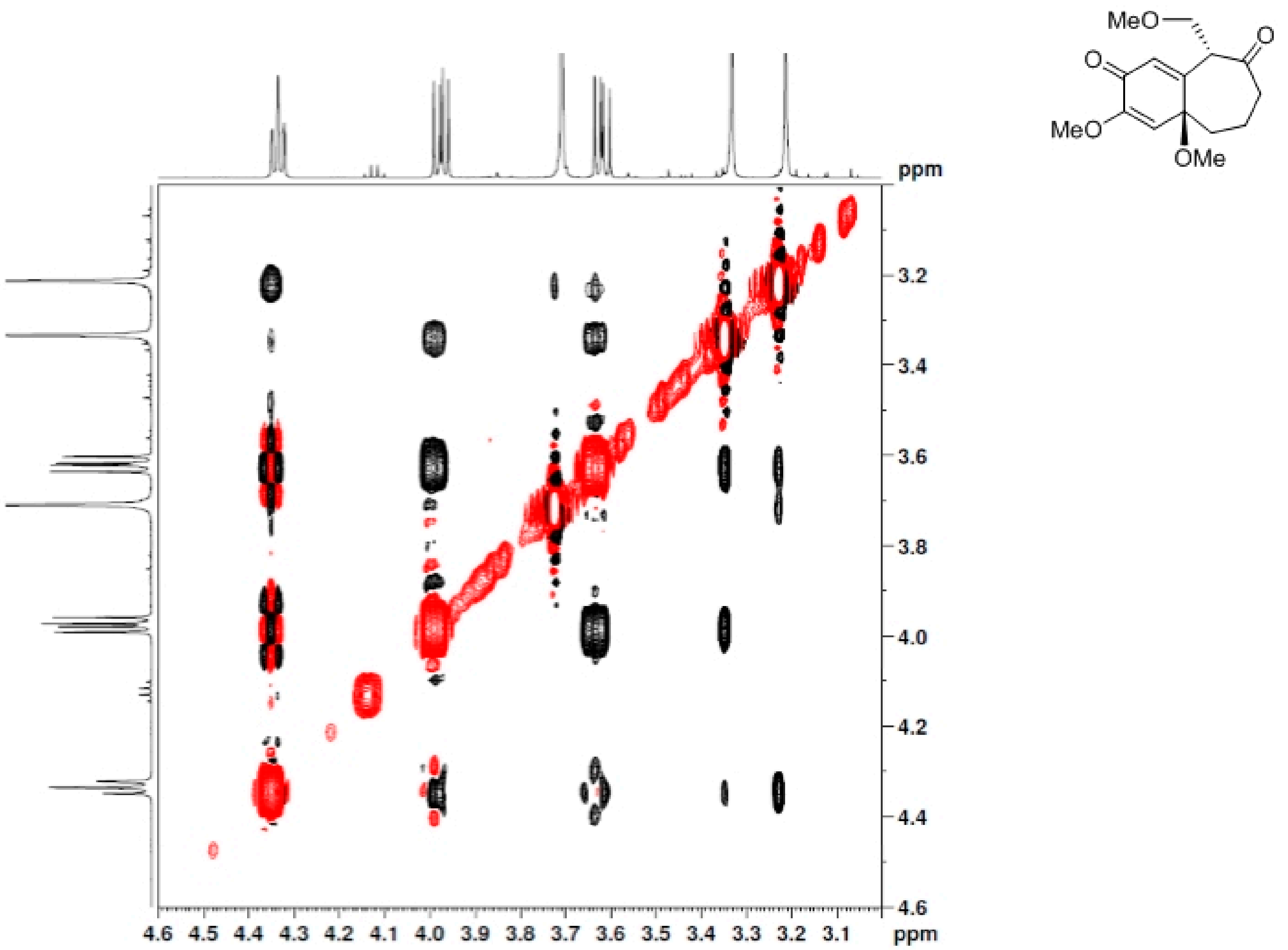

Figure S21. Expansion of ^{1}H-^{1}H NOESY spectra of *trans*-3,4a-dimethoxy-9-(methoxymethyl)-4a,5,6,7-tetrahydro-2*H*-benzo[7]annulene-2,8(9*H*)-dione (**12f**) (500 MHz, $CDCl_3$).

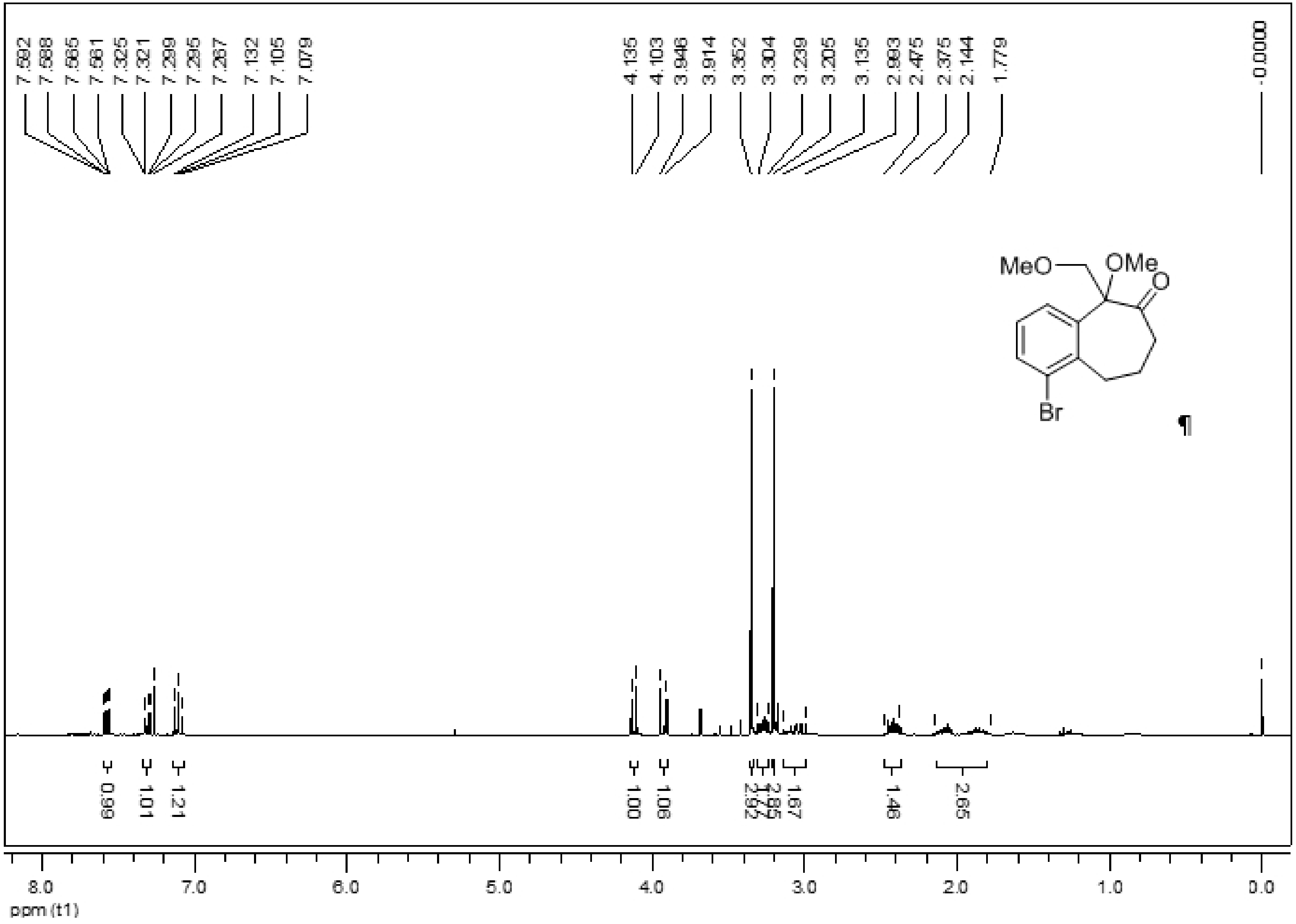

Figure S22. ^{1}H-NMR spectra of 1-bromo-5-methoxy-5-(methoxymethyl)-5,7,8,9-tetrahydro-6*H*-benzo[7]annulen-6-one (**8g**) (300 MHz, $CDCl_3$).

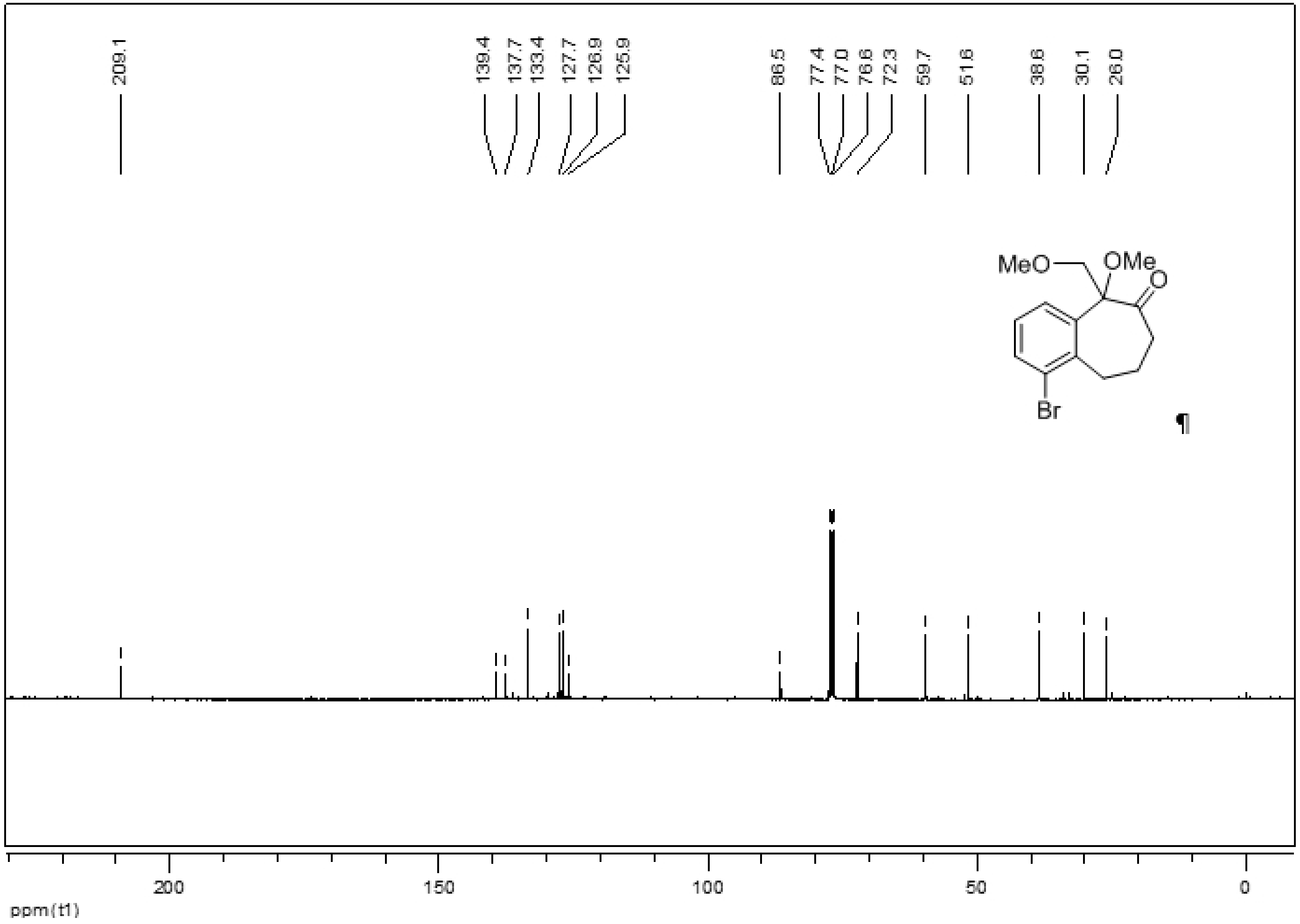

Figure S23. ^{13}C-NMR spectra of 1-bromo-5-methoxy-5-(methoxymethyl)-5,7,8,9-tetrahydro-6*H*-benzo[7]annulen-6-one (**8g**) (75 MHz, $CDCl_3$).

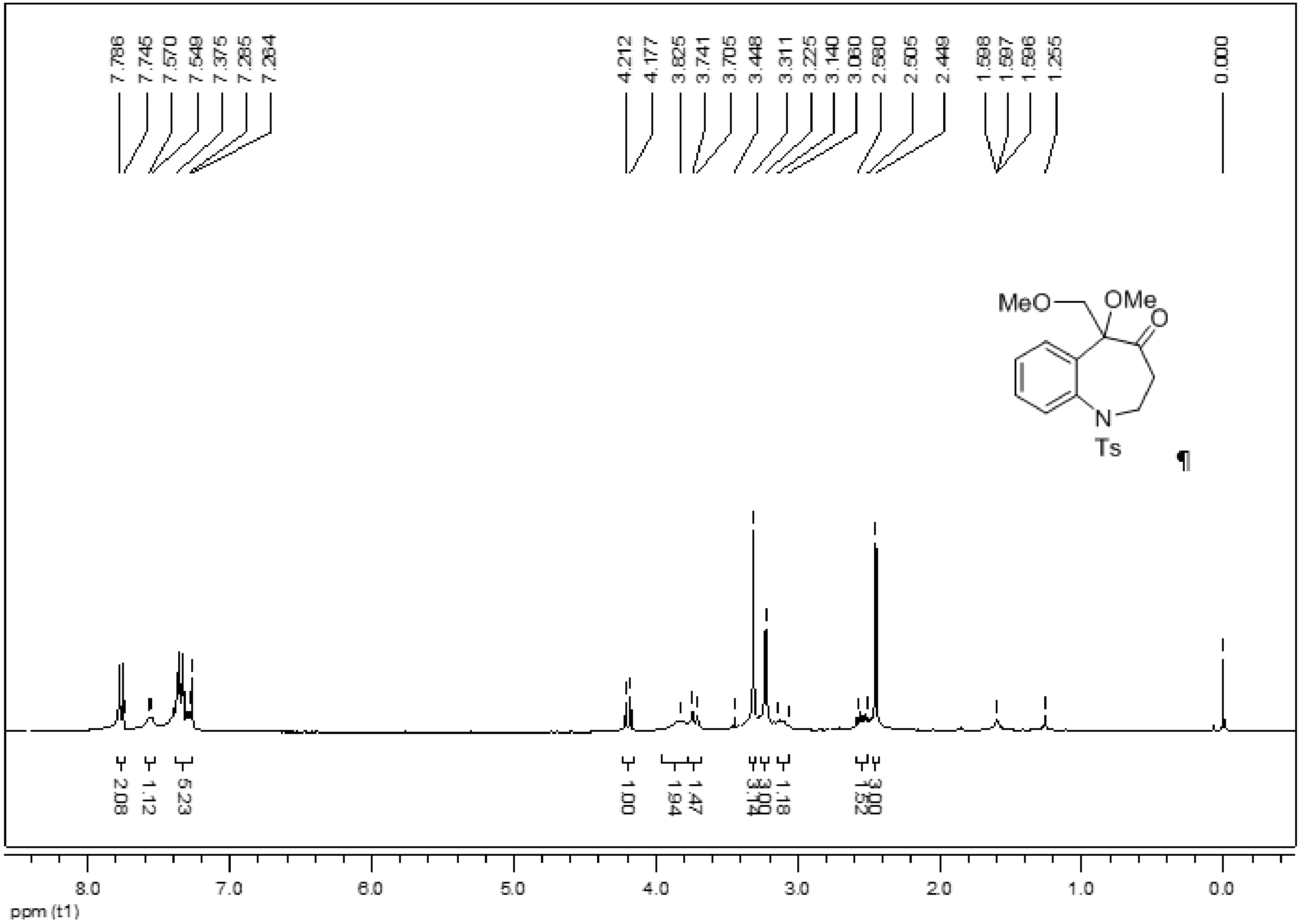

Figure S24. ^{1}H-NMR spectra of 5-methoxy-5-(methoxymethyl)-1-tosyl-1,2,3,5-tetrahydro-4*H*-benzo[*b*]azepin-4-one (**8l**) (300 MHz, $CDCl_3$).

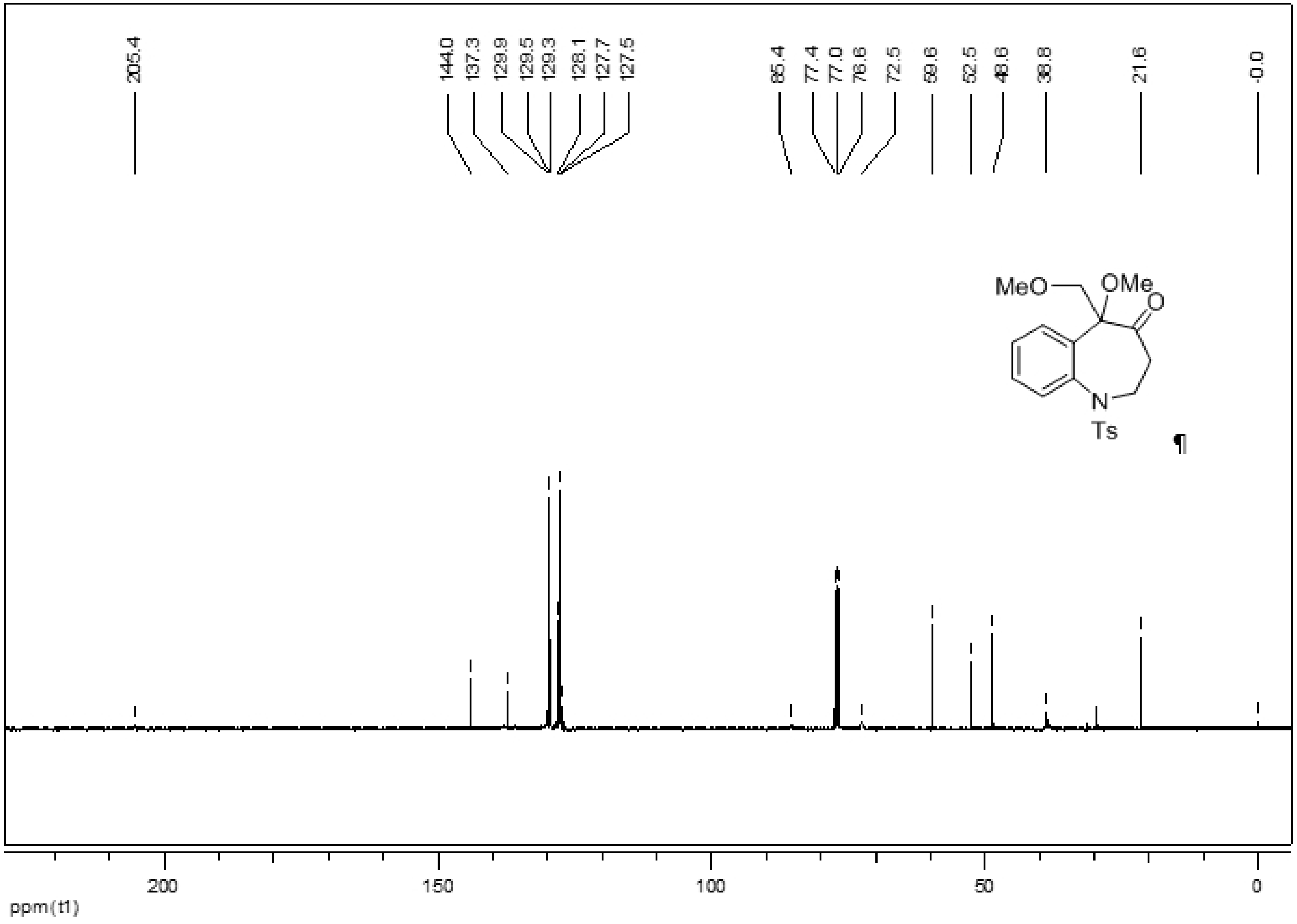

Figure S25. ^{13}C-NMR spectra of 5-methoxy-5-(methoxymethyl)-1-tosyl-1,2,3,5-tetrahydro-4*H*-benzo[*b*]azepin-4-one (**8l**) (75 MHz, $CDCl_3$).

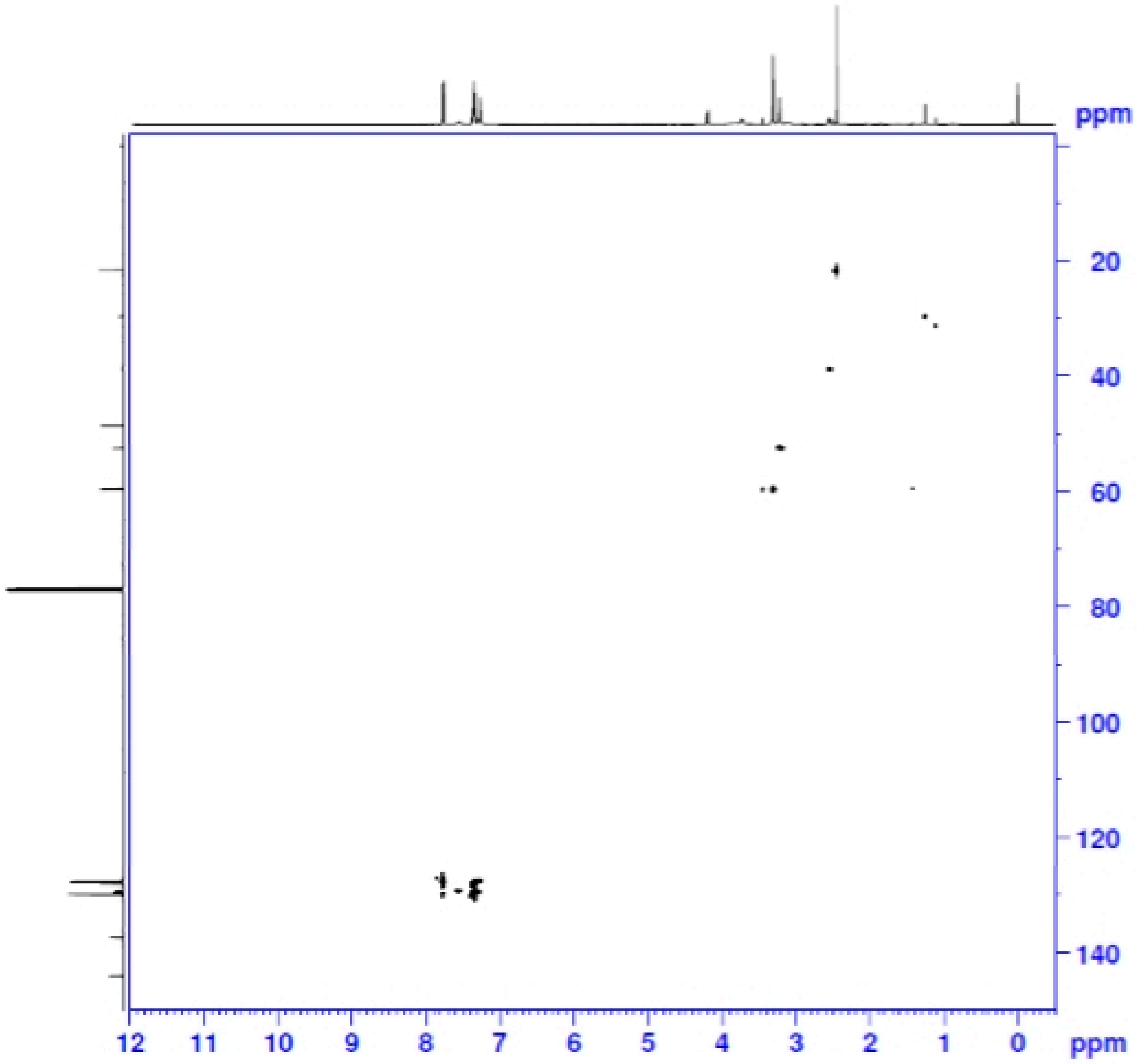

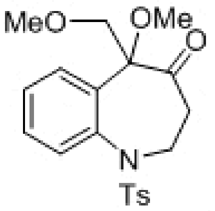

Figure S26. ^{1}H-^{13}C HSQC spectra of 5-methoxy-5-(methoxymethyl)-1-tosyl-1,2,3,5-tetrahydro-4*H*-benzo[*b*]azepin-4-one (**8l**) (500 MHz, $CDCl_3$).

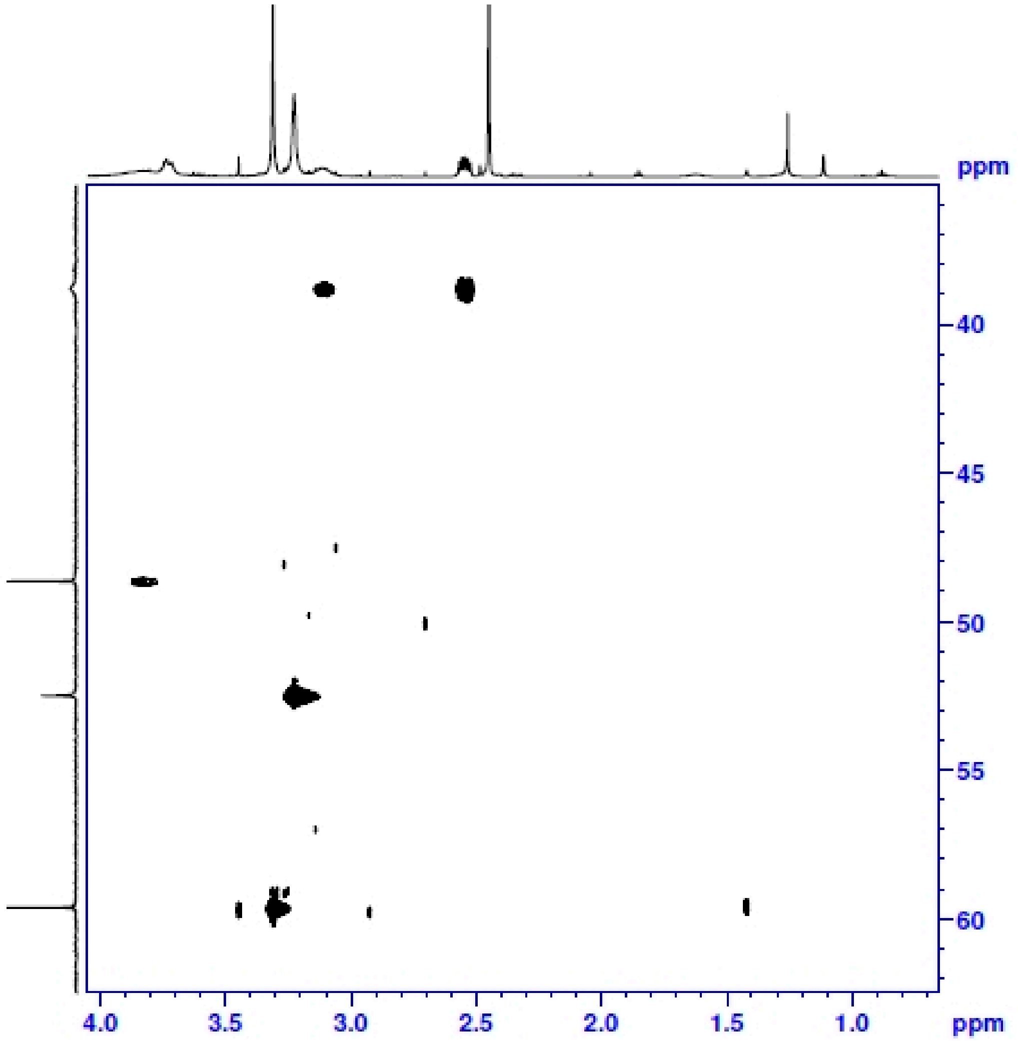

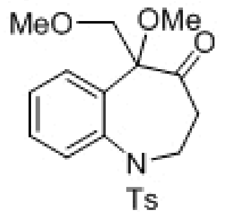

Figure S27. First Expansion of ^{1}H-^{13}C HSQC spectra of 5-methoxy-5-(methoxymethyl)-1-tosyl-1,2,3,5-tetrahydro-4*H*-benzo[*b*]azepin-4-one (**8l**) (500 MHz, $CDCl_3$).

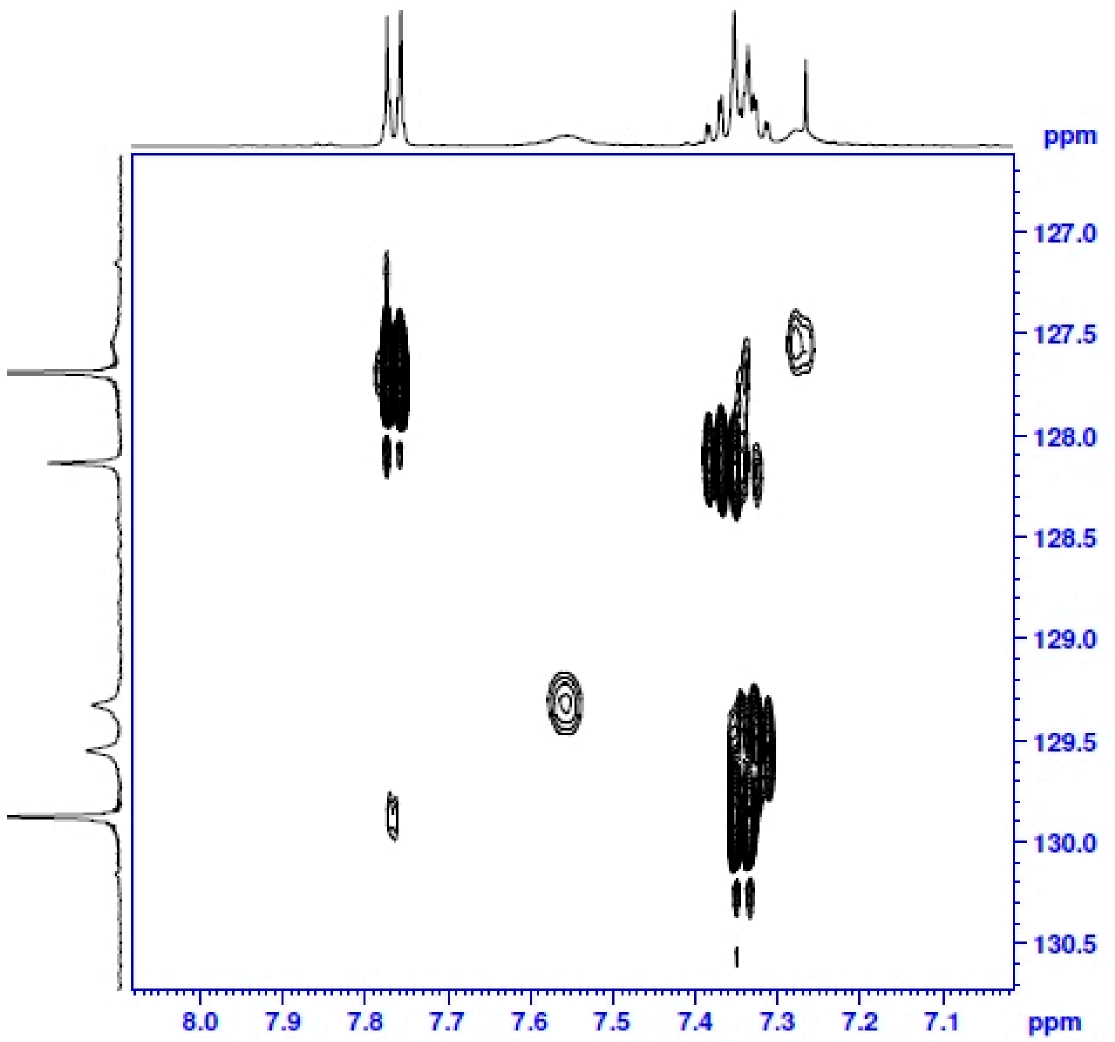

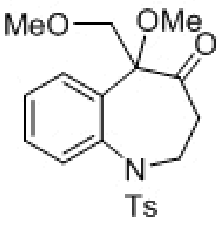

Figure S28. Second Expansion of ¹H-¹³C HSQC spectra of 5-methoxy-5-(methoxymethyl)-1-tosyl-1,2,3,5-tetrahydro-4*H*-benzo[*b*]azepin-4-one (**8l**) (500 MHz, $CDCl_3$).

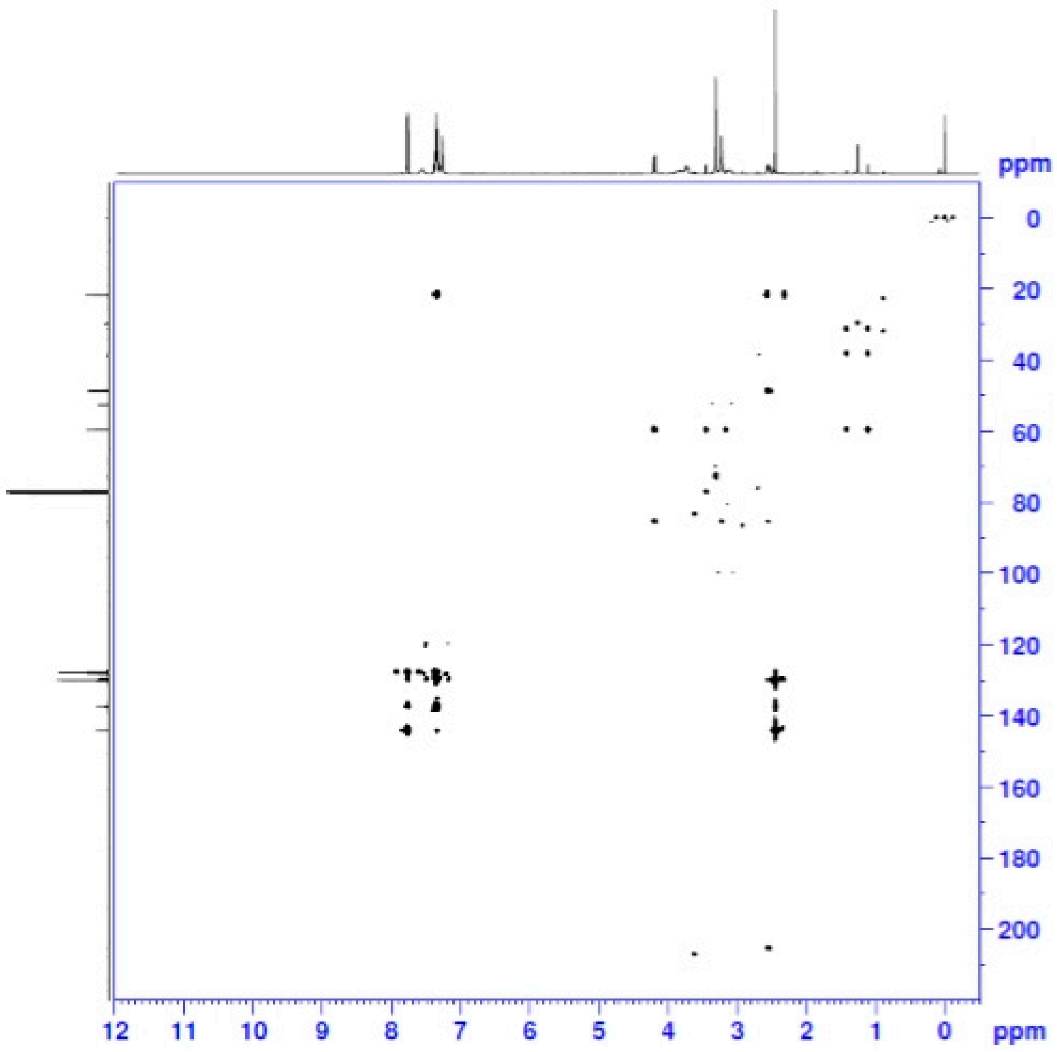

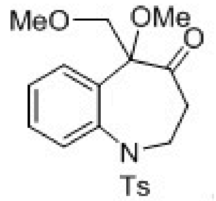

Figure S29. ^{1}H-^{13}C HMBC spectra of 5-methoxy-5-(methoxymethyl)-1-tosyl-1,2,3,5-tetrahydro-4*H*-benzo[*b*]azepin-4-one (**8l**) (500 MHz, $CDCl_3$).

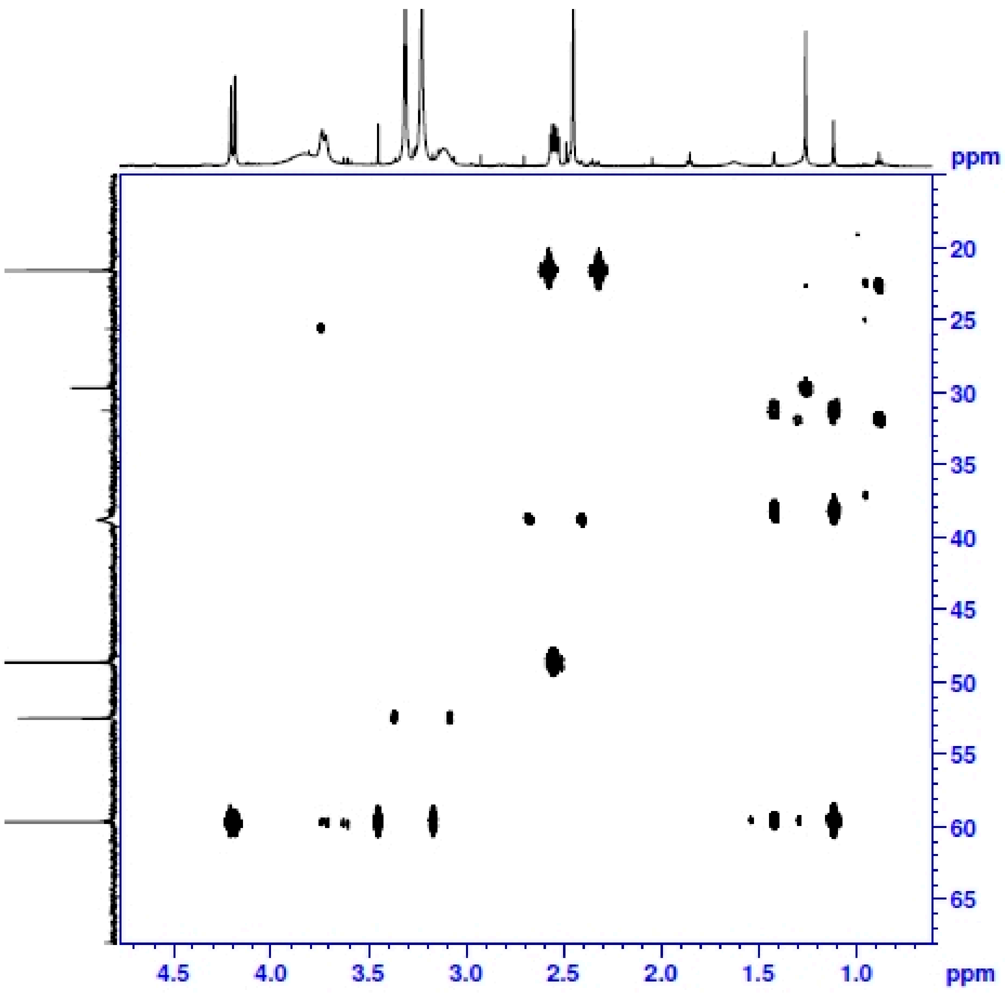

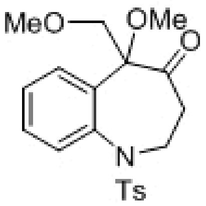

Figure S30. First Expansion of ^{1}H-^{13}C HMBC spectra of 5-methoxy-5-(methoxymethyl)-1-tosyl-1,2,3,5-tetrahydro-4*H*-benzo[*b*]azepin-4-one (**8l**) (500 MHz, CDCl$_3$).

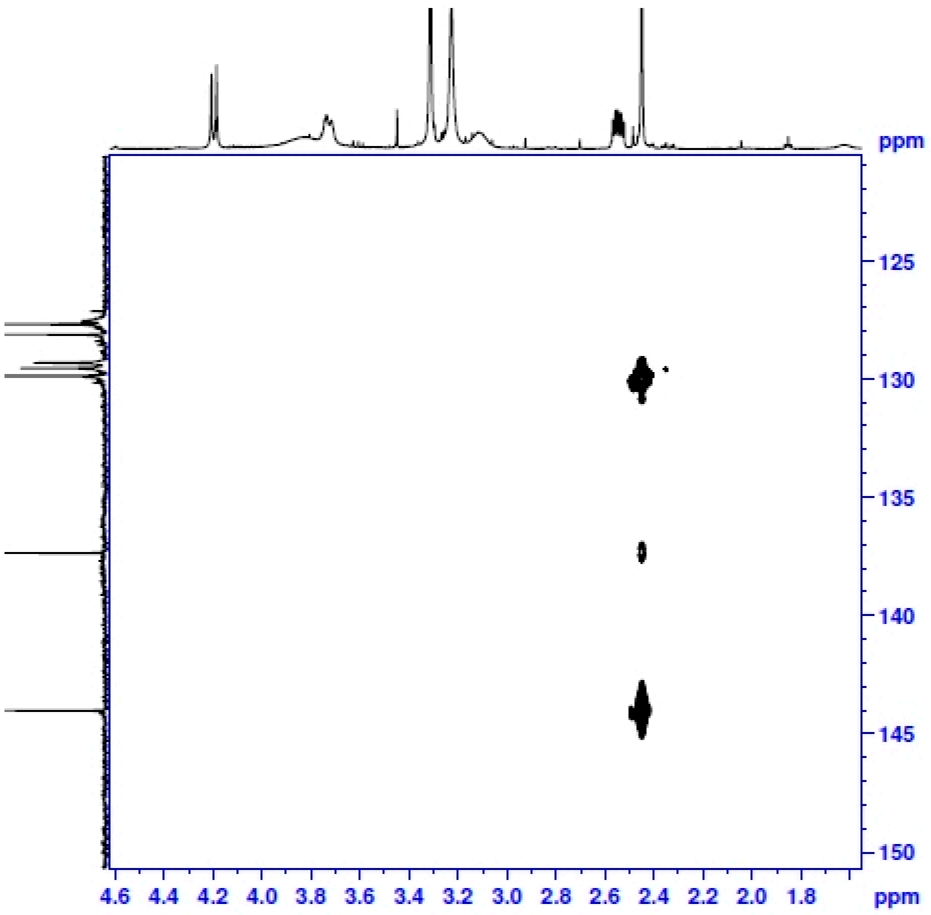

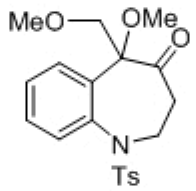

Figure S31. Second Expansion of ^{1}H-^{13}C HMBC spectra of 5-methoxy-5-(methoxymethyl)-1-tosyl-1,2,3,5-tetrahydro-4*H*-benzo[*b*]azepin-4-one (**8l**) (500 MHz, $CDCl_3$).

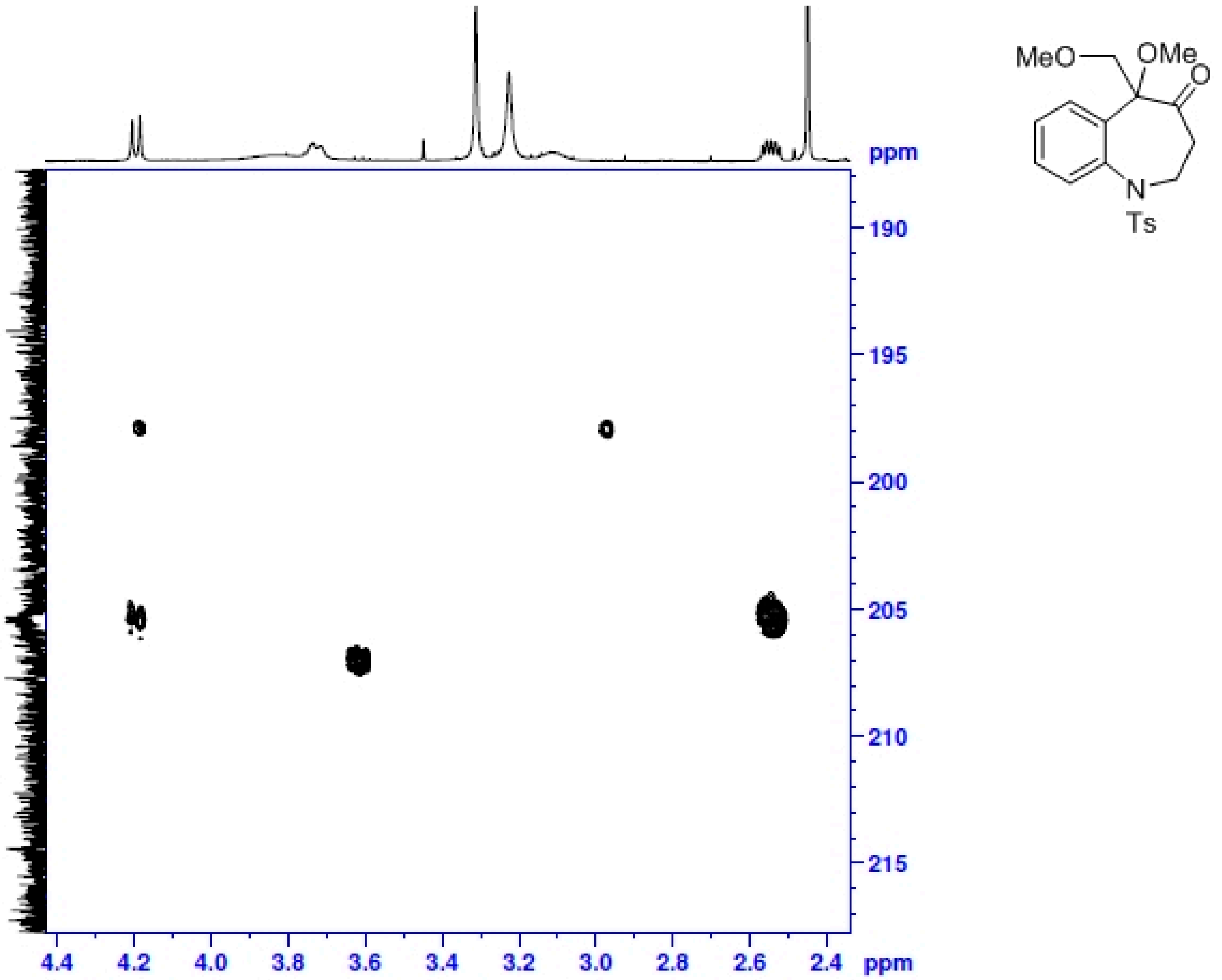

Figure S32. Third Expansion of ^{1}H-^{13}C HMBC spectra of 5-methoxy-5-(methoxymethyl)-1-tosyl-1,2,3,5-tetrahydro-4*H*-benzo[*b*]azepin-4-one (**8l**) (500 MHz, $CDCl_3$).

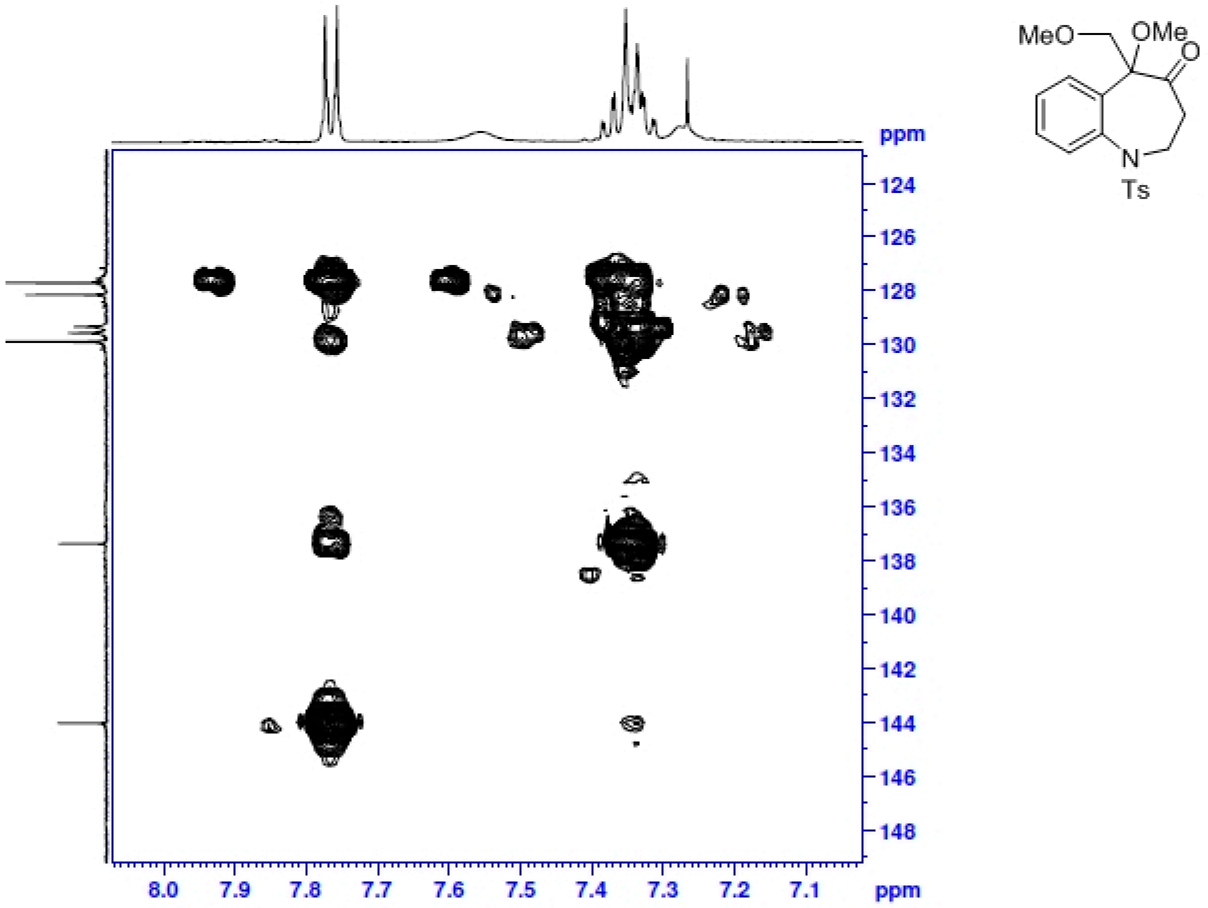

Figure S33. Fourth Expansion of ¹H-¹³C HMBC spectra of 5-methoxy-5-(methoxymethyl)-1-tosyl-1,2,3,5-tetrahydro-4*H*-benzo[*b*]azepin-4-one (**8l**) (500 MHz, $CDCl_3$).
